# Inerter-Based Suspension for Wheel Lift-Off Suppression of Manned Lunar Rovers

**DOI:** 10.3390/s26217074

**Published:** 2026-11-09

**Authors:** Yanling Liu, Hong Wang, Yujie Shen, Xiaofeng Yang, Changzhuang Chen

**Affiliations:** 1 School of Automotive and Traffic Engineering, Jiangsu University, Zhenjiang 212013, China; liuyl@ujs.edu.cn (Y.L.); wh13056095846@163.com (H.W.); yangxf18@ujs.edu.cn (X.Y.); cchangzhuang@163.com (C.C.); 2 Automotive Engineering Research Institute, Jiangsu University, Zhenjiang 212013, China

**Keywords:** manned lunar rover, wheel lift-off, inerter suspension, wheel–ground contact, parameter optimization

## Abstract

Wheel lift-off under low gravity and rough terrain can degrade ride comfort and wheel–ground contact of manned lunar rovers. This study investigates whether an inerter-based suspension can improve vertical dynamics and contact retention. A Bekker-based equivalent wheel–regolith model was linearized around static equilibrium and incorporated into a dimensionless piecewise quarter-vehicle model with contact/separation switching. Parallel and series inerter–damper configurations were compared using frequency-response, lift-off-boundary, and time-domain analyses, and suspension parameters were optimized for rough-mare and rough-upland excitations. The series configuration exhibited a larger contact region and lower off-ground time over the investigated parameter range. After optimization, body-acceleration RMS decreased by 19.61% and 15.66%, contact-force RMS by 13.75% and 13.67%, off-ground time ratio by 59.5% and 24.5%, and lift-off counts by 42.8% and 45.5% for rough mare and rough upland, respectively, relative to the conventional suspension. These results show that appropriate inertance–damping matching can improve vertical vibration performance and wheel-contact retention under the prescribed lunar-terrain conditions.

## 1. Introduction

The manned lunar rover serves as the core equipment for human–robot collaborative exploration on the lunar surface [1,2]. Its primary functions not only involve supporting astronauts in their mobility and operations on the Moon but also include carrying scientific instruments for environmental monitoring and data collection [3]. However, the gravitational force on the Moon is only about one-sixth that of Earth’s, coupled with the frequent occurrence of low-frequency, high-amplitude impact excitations [4]. This combination increases the likelihood of the rover’s wheels becoming airborne [5], which significantly affects the safety of astronauts and their riding comfort, thereby jeopardizing the successful execution of lunar exploration missions [6]. Consequently, effectively suppressing wheel lift-off has emerged as a pressing issue that needs to be addressed in the structural design and development of manned lunar rovers [7,8]. More generally, terrain-aware planning for off-road vehicles combines terrain characteristics with vehicle traversing capability to identify passable regions and select feasible paths [9]. Recent chassis studies have further emphasized that mobility and terrain adaptability depend on the coordinated interaction among chassis configuration, terrain conditions, and operating requirements [10].

The suspension is the key subsystem that connects the chassis and the wheels [11,12]. It governs the vertical dynamic response of a crewed lunar rover and the distribution of dynamic wheel loads [13]. It is also central to suppressing wheel–ground separation and maintaining continuous ground contact [3]. Existing studies have examined this issue from the perspectives of suspension architecture, parameter matching, and dynamic optimization. Chen et al. investigated structural vibration under multi-source excitation and showed that the coupling between multiple excitation sources and structural modes can significantly affect the dynamic response of a vehicle system [14]. Wang et al. developed a quarter-vehicle model incorporating rigid-wheel–terrain interaction to investigate vertical vibration on deformable terrain [5]. Their formulation assumed continuous wheel–ground contact. Liu et al. subsequently investigated contact and separation using switching pitch-dynamics models, with off-ground time and pitch-angle variation as stability indicators [3]. Zhou et al. developed an electromechanical active suspension and evaluated its performance under simulated lunar gravity and rough-terrain conditions [6]. More recently, Zhou et al. investigated wheel–ground-clearance reduction through active suspension control [15]. These studies provide relevant foundations for examining suspension performance during wheel–ground contact and separation. For heavy multi-wheeled lunar vehicles, Hu et al. proposed a parallel wheelset suspension and showed through simulation that this design can improve ride comfort and enhance wheel–ground contact [16]. Cui et al. optimized suspension parameters under dynamic excitation and verified the resulting vibration-reduction performance through a six-degree-of-freedom motion simulation and experimental platform [17]. Subsequently, to address the issue of poor ground contact between the wheels and the ground when the parallel wheelset suspension system traverses rough terrain, the suspension system was optimized to improve its straight-line stability [18]. Recent suspension studies have addressed load uncertainty and nonlinear damping constraints using magnetorheological suspension control and have coordinated ride-comfort and suspension-deflection objectives in fault-tolerant active suspension control [19,20]. These studies highlight the importance of considering operating-condition variations and competing performance requirements in suspension design.

In addition, the ground-simulation study by Yuan et al. showed that changes in the gravity field can significantly alter vehicle body responses in bounce, pitch, and roll for lunar vehicles with damping elements [21]. From the perspective of parameter design, Zhang et al. developed a bi-stage optimization framework for crewed lunar rovers [22]. Their framework incorporated elastic wheel-terrain interaction and a deep-neural-network surrogate model for suspension parameter optimization, demonstrating the relevance of coordinated parameter design to lunar-rover performance. Chen et al. further showed that low gravity and rugged terrain can induce wheel bounce and that the matching of hardpoint and wheel-alignment parameters in a double-wishbone suspension affects wheel-posture stability and operational reliability [23]. For ground vehicles operating on rough terrain, attitude-constrained trajectory planning has also been investigated to reduce roll and pitch responses [24]. Hu et al. developed an adaptive chassis-leveling system for uneven terrain, demonstrating that active attitude adjustment can effectively compensate for longitudinal and lateral inclination induced by terrain disturbances [25]. He further analyzed the dynamic stress response of an attitude-adjustable chassis and showed that terrain-induced attitude variation and chassis adjustment can significantly influence the dynamic loads of key structural components [26]. Overall, existing studies indicate that appropriate suspension architecture and parameter matching can improve the vertical dynamic response and wheel–ground contact performance of lunar rovers. Xu et al. demonstrated that low-frequency vertical vibration is an important contributor to ride discomfort and that appropriate damping and structural modification can effectively reduce vibration transmission [27]. Gao et al. employed time- and frequency-domain analyses to identify the dominant vibration components and transmission characteristics of a vehicle-terrain system underground excitation [28]. Under lunar low-gravity conditions, rough-terrain excitation imposes competing requirements on vibration isolation, suspension travel, and wheel–ground contact retention [6]. Inerter-based suspensions provide additional flexibility in shaping the dynamic response through their connection topology and inertance parameters [29]; examining how these design variables affect wheel lift-off can therefore provide useful guidance for suspension parameter selection under lunar operating conditions.

An inerter is a two-terminal mechanical element whose force is proportional to the relative acceleration between its terminals [30]. Inerter-based designs have been investigated in vehicle and seat suspension systems [31,32,33,34]. Li et al. examined a two-stage inerter-spring-damper suspension under random and periodic excitation, analyzing the effects of inertance on vibration performance and resonance suppression [32]. Zhang et al. compared eight passive and semi-active suspension layouts using a quarter-car model, evaluating sprung-mass acceleration, suspension travel, and tire deflection [33]. These studies demonstrate the importance of network layout and parameter selection in coordinating vibration isolation and road-holding performance.

Zhang et al. also showed that ISD suspensions reduce low-frequency resonance peaks and the root mean square of body acceleration, demonstrating their potential for low-frequency vibration suppression and improved isolation [34]. Wang et al. investigated damping optimization under multi-source excitation and showed that identifying dominant vibration-transmission paths provides an effective basis for reducing structural dynamic response [35]. Sun et al. combined multi-objective optimization with signal-decomposition techniques for load-spectrum reconstruction, demonstrating the applicability of multi-objective optimization to vibration-related parameter design under complex excitation [36]. Dai et al. constructed representative ground-vibration load spectra from measured signals using signal decomposition, statistical extrapolation, and spectral analysis, providing a method for characterizing complex random ground excitation [37]. Yang et al. combined time- and frequency-domain information from vehicle operating data to estimate tractor–trailer mass and validated the method through real-vehicle experiments [38]. Chen et al. further integrated measurable chassis-state information with real-time predictive control and validated the control strategy on a real-vehicle platform, highlighting the importance of state perception and model-based decision making in intelligent chassis systems [39]. 

The studies above establish relevant foundations in lunar-rover contact-separation dynamics [3,5], active suspension control [6,15], suspension parameter optimization [22], and inerter-based vibration reduction [32,33]. Building on these results, a specific design question concerns how passive series and parallel inerter–damper networks affect the onset and duration of wheel lift-off under lunar gravity. Clarifying this relationship requires evaluating topology and inertance-damping matching together with the transitions between wheel–ground contact and separation.

Accordingly, this study compares conventional, parallel inerter–damper, and series inerter–damper suspensions within a dimensionless piecewise quarter-vehicle framework. Lift-off boundaries and time-domain contact responses are examined to characterize topology-dependent inertance effects, including the non-monotonic variation of off-ground time in the series configuration. The selected series configuration is then optimized under prescribed rough-mare and rough-upland excitations, with vibration response, off-ground time ratio, and lift-off count used to evaluate the resulting performance. 

The main contributions of this study are summarized as follows:

(1) The influence of wheel–ground separation on suspension response under lunar gravity is quantified by comparing predictions with and without contact-separation switching, highlighting its importance in evaluating vertical vibration and wheel-contact performance.
(2) The effects of parallel and series inerter–damper configurations on lift-off boundaries and contact-force responses are characterized. The non-monotonic variation of off-ground time with inertance in the series configuration is identified, providing a basis for inertance selection.
(3) Inertance and damping are optimized for the selected series configuration under prescribed rough-mare and rough-upland excitations. The resulting changes in body acceleration, wheel–ground contact force, off-ground time ratio, and lift-off count are evaluated against a conventional suspension baseline.

The structure of the remaining sections of this article is as follows: The Section 2 presents the establishment of the wheel–ground contact model, along with the development and dimensionless formulation of the dynamics model for the manned lunar rover considering wheel lift-off under both inerter–damper series and parallel configurations; The Section 3 conducts a study on the wheel lift-off response under both inerter–damper series and parallel configurations, and establishes the boundary diagram of wheel–ground separation; The Section 4 establishes the low-frequency large-amplitude excitation model for the lunar surface and optimizes the parameters of the manned lunar rover’s inerter suspension system under two types of excitations using a multi-objective particle swarm optimization algorithm; The Section 5 presents the conclusions.

## 2. Establishment of the Dynamic Model of the Inerter Suspension System for the Manned Lunar Rover

### 2.1. Dynamic Model of Wheel–Regolith Interaction

Radial deformation of an elastic wheel changes the geometry of its contact region with the lunar regolith. To describe the deformed contact geometry, an equivalent rigid wheel is introduced for calculating the normal stress distribution and vertical supporting load. The equivalent wheel radius is expressed as

(1) $$r_{v}=r\sin(\frac{\theta_{1}+\theta_{2}}{2})/\sin(\frac{\theta_{1}-\theta_{2}}{2})$$

where $r_{v}$ is radius of the virtual rigid wheel, r is radius of the original elastic wheel, $\theta_{1}$ is the entry angle, and $\theta_{2}$ is the exit angle.

**Figure 1:**
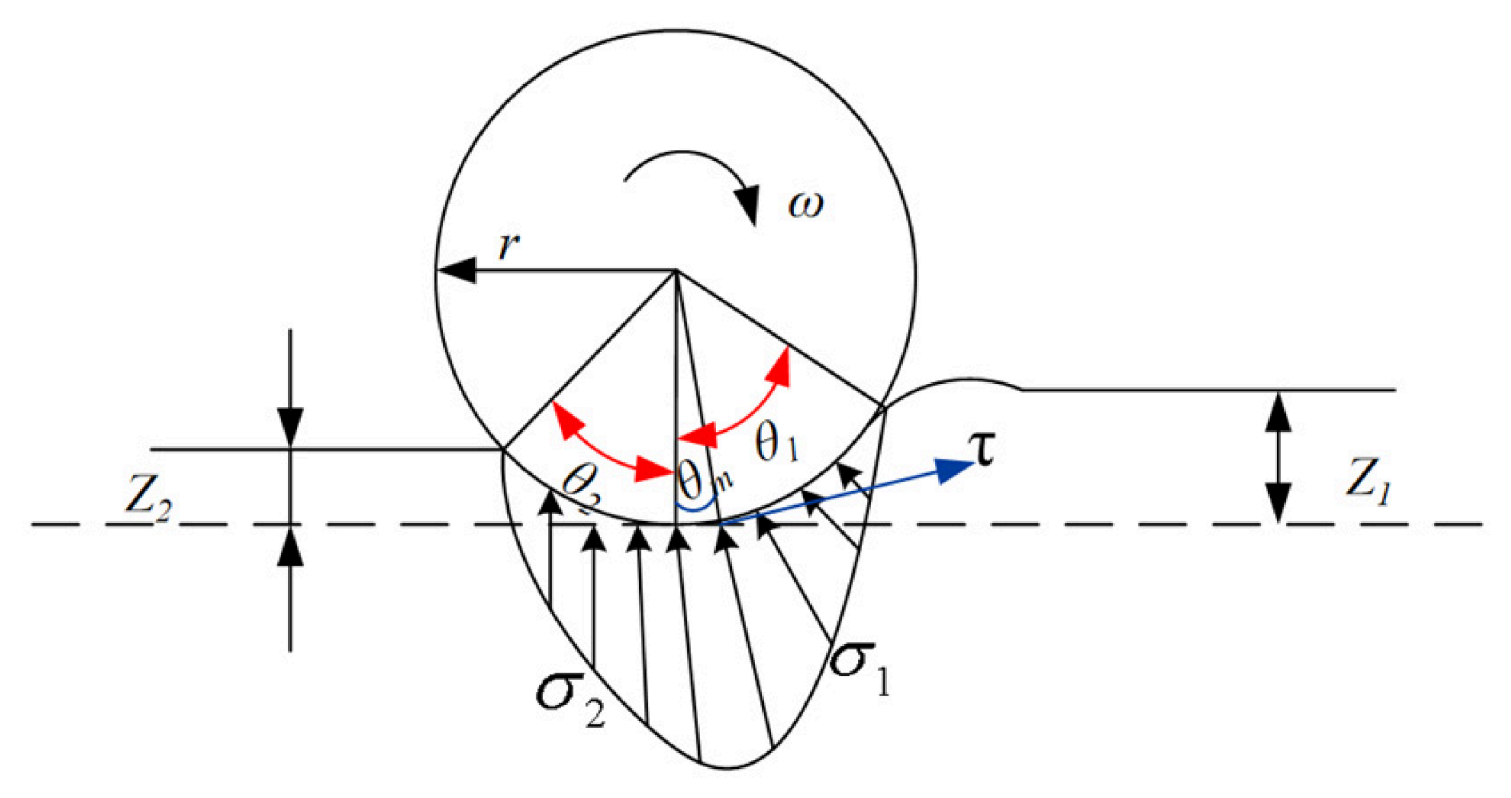
Wheel–regolith interaction analysis diagram.

In Figure 1, $z_{1}$ denotes the wheel sinkage, and $z_{2}$ denotes the soil rebound height behind the wheel [40]. Both heights are measured from the horizontal dashed line. The ground surface behind the wheel remains lower than that ahead of the wheel by $z_{1}-z_{2}$, indicating residual sinkage after wheel passage.

Therefore, the contact between the elastic wheel and soft lunar regolith is represented by an equivalent rigid-wheel model, allowing for the normal stress distribution and vertical supporting force to be evaluated using the pressure–sinkage relationship.

Based on the interaction between an equivalent rigid wheel and soft lunar regolith, the soil settlement z(θ) can be used to describe the local subsidence of the terrain within the contact area. Since the soft lunar regolith exhibits nonlinear pressure-settlement behavior under wheel loading, the Bekker pressure–sinkage model is introduced to establish the relationship between the soil settlement z(θ) and the normal stress σ(θ) at the contact interface. The soil settlement z(θ) and the normal stress σ(θ) are given as follows:

(2) $$z(\theta )=r_{v}(\cos\theta -\cos\theta_{1})$$

(3) $$\sigma (\theta )=\left(\frac{K_{c}}{B}+K_{\varphi }\right)z{\left(\theta \right)}^{n}$$

The regolith parameters are taken as spatially uniform within each terrain case [41], with their values listed in Table 1.

When the wheel comes into contact with the soft lunar soil, the stress distribution along the contact arc is asymmetrical, with the maximum stress occurring at $\theta_{m}$, where $\theta_{m}$ denotes the angle of maximum stress. The normal stress distribution in the two parts of the contact region is described by the Wong-Reece formulation based on the Bekker pressure–sinkage relationship [40]:

(4) $$\left\{\begin{array}{cc} \sigma_{1}(\theta )=(\frac{K_{c}}{B}+K_{\varphi }){r_{v}}^{n}{\left(\cos\theta -\cos\theta_{1}\right)}^{n} & \left(\theta_{m}\leq \theta \leq \theta_{1}\right)\\ \sigma_{2}(\theta )=(\frac{K_{c}}{B}+K_{\varphi }){r_{v}}^{n}{\left(\cos\left(\theta_{1}-\frac{\theta -\theta_{2}}{\theta_{m}-\theta_{2}}\left(\theta_{1}-\theta_{m}\right)\right)-\cos\theta_{1}\right)}^{n} & \left(\theta_{2}\leq \theta \leq \theta_{m}\right) \end{array}\right.$$

The regolith stiffness is determined from the normal load–sinkage relation about static equilibrium under vertical wheel loading. Small variations in the applied load produce corresponding changes in sinkage, and the local slope of this relation defines the tangent stiffness [41]. The normal supporting load is obtained by integrating the vertical component of the normal pressure over the wheel–regolith contact region:

(5) $$W=Br_{v}\left(\int_{\theta_{2}}^{\theta_{m}}\sigma_{2}(\theta )\cos\theta d\theta +\int_{\theta_{m}}^{\theta_{1}}\sigma_{1}(\theta )\cos\theta d\theta \right)$$

The obtained vertical load can be expressed as a nonlinear function of the wheel sinkage, and the corresponding implicit equilibrium equation is written as:

(6) F(W,z)=W−f(z)=0

At the static equilibrium point $\left(W_{0},z_{0}\right)$,

(7) $$F(W_{0},z_{0})=0$$

By performing a first-order Taylor expansion of F(W,z) around $\left(W_{0},z_{0}\right)$ and neglecting higher-order terms, the load–sinkage relationship can be linearized as:

(8) $$W-W_{0}=k_{1}(z-z_{0})$$

where $k_{1}$ is the tangent wheel–regolith sinkage stiffness at the static equilibrium point. 

Following the stiffness-equivalence procedure described by Cheng [41], the tangent stiffness of the regolith at static equilibrium is combined in series with the radial stiffness of the elastic wheel. The resulting equivalent vertical stiffness accounts for the contributions of wheel deformation and regolith sinkage and is expressed as

(9) $$k_{t}=\frac{k_{1}k_{2}}{k_{1}+k_{2}}$$

where $k_{2}$ is the vertical stiffness of the original elastic wheel.

**Figure 2:**
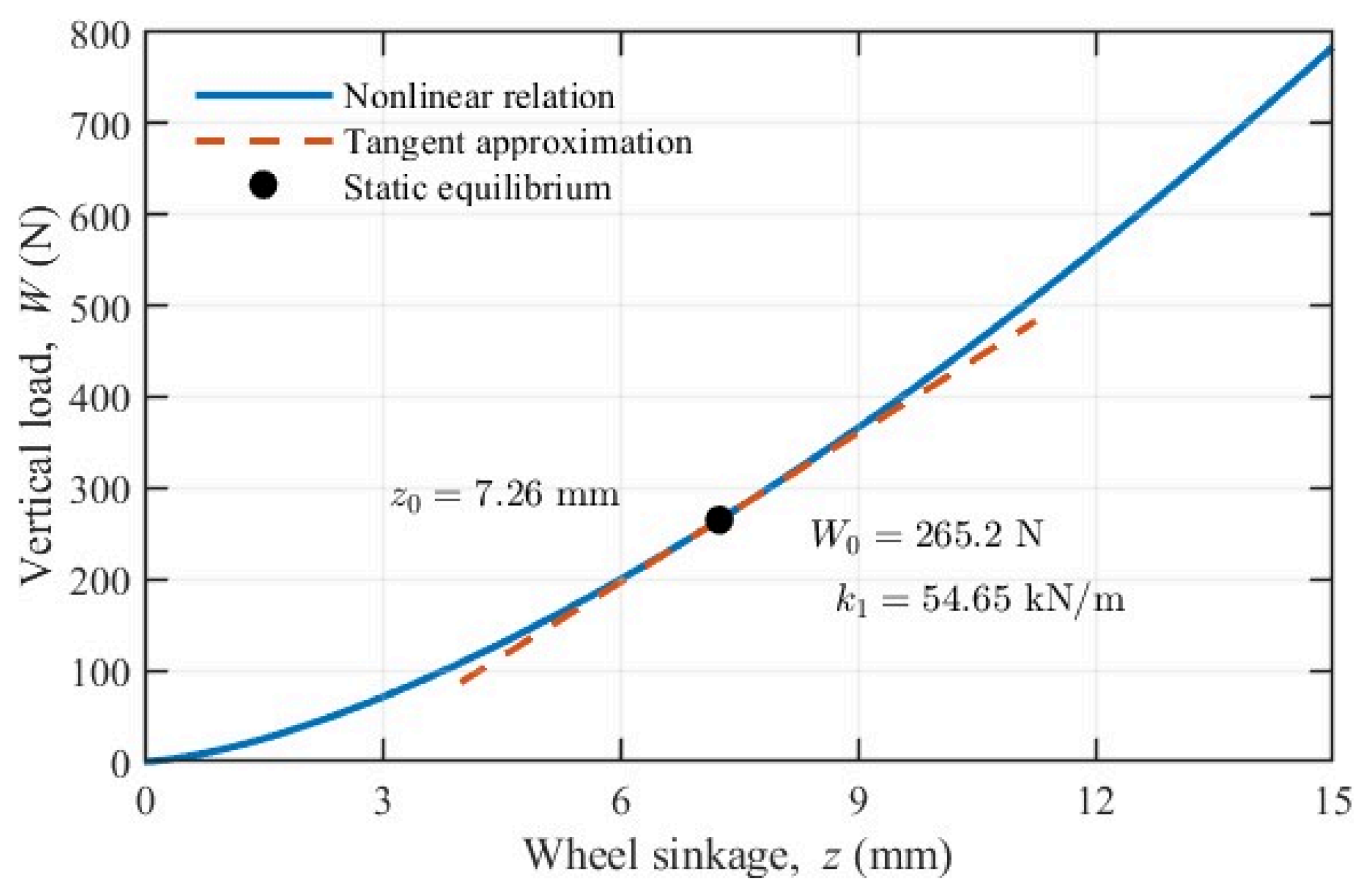
Nonlinear wheel–regolith load–sinkage relation and its local tangent approximation at the static equilibrium point.

Figure 2 compares the nonlinear normal load–sinkage relation with its tangent approximation at the static equilibrium point to assess the local linearization used in the stiffness calculation. The static equilibrium occurs at $z_{0}=7.26\;\mathrm{mm}$ with a vertical load of $W_{0}=265.2\;N$. The corresponding tangent sinkage stiffness is $k_{1}=54.65$ kN/m. After considering the series combination with the elastic-wheel stiffness, the corrected equivalent vertical stiffness is $k_{t}=16.404$ kN/m. Within the selected neighborhood of the static operating point, the maximum deviation between the nonlinear relation and its tangent approximation is approximately 6.38%. Within the illustrated sinkage range, the tangent approximation closely follows the nonlinear load–sinkage relation, supporting the use of the corresponding tangent stiffness to describe the local normal response of the regolith around static equilibrium.

### 2.2. Establishment of the Dynamic Model

In the low-gravity environment of the lunar surface, the manned lunar rover experiences significant low-frequency, high-amplitude road excitations during operation. Compared to ground vehicles, manned lunar rovers are more prone to wheel lift-off events, rendering the assumption of constant wheel–ground contact in traditional quarter-vehicle models no longer valid. 

Therefore, it is necessary for the quarter-vehicle dynamic model established for manned lunar rovers to account for wheel lift-off phenomena in order to analyze the critical frequency at which wheel lift-off occurs, the system’s frequency response, and their impact on suspension structure selection and parameter design.

**Figure 3:**
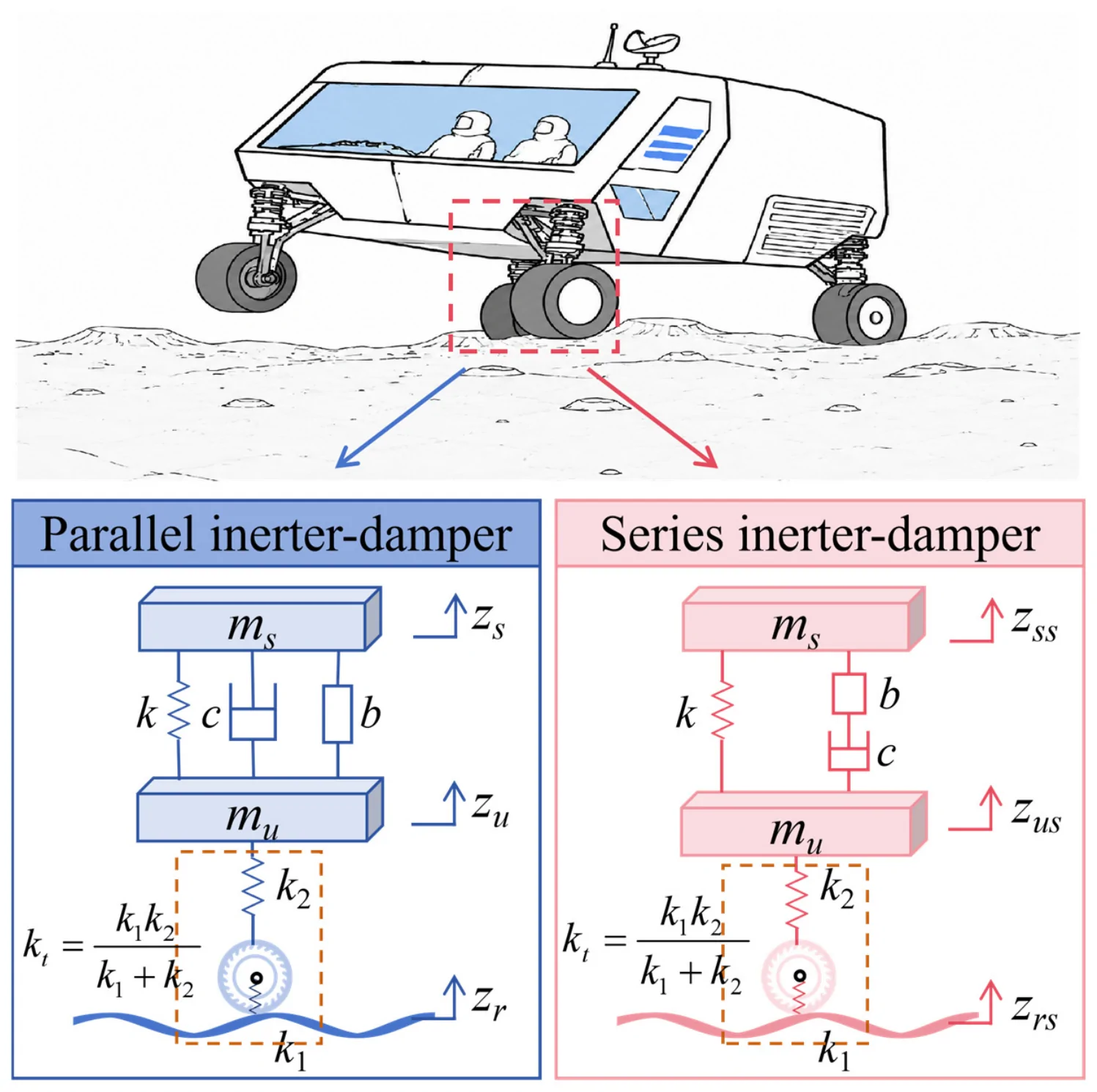
Parallel inerter–damper configuration and series inerter–damper configuration.

After the corrected equivalent vertical stiffness $k_{t}$ of the elastic wheel–regolith system is obtained, it is introduced into the vertical dynamic model of the manned lunar rover. To facilitate the subsequent analysis of wheel lift-off characteristics under low-frequency, large-amplitude excitation, quarter-vehicle models with inerter–damper parallel and series configurations are established, as shown in Figure 3.

For the parallel inerter–damper suspension, the suspension spring, damper, and inerter are arranged in parallel between the sprung and unsprung masses. Considering the static deflection of the suspension and the static compression of the equivalent wheel–regolith system, the general governing equations can be written as

(10) $$\left\{\begin{array}{l} m_{s}{\ddot{z}}_{s}+k(z_{s}-z_{u}-l_{1})+c({\dot{z}}_{s}-{\dot{z}}_{u})+b({\ddot{z}}_{s}-{\ddot{z}}_{u})+m_{s}g_{m}=0\\ m_{u}{\ddot{z}}_{u}-k(z_{s}-z_{u}-l_{1})-c({\dot{z}}_{s}-{\dot{z}}_{u})-b({\ddot{z}}_{s}-{\ddot{z}}_{u})+k_{t}(z_{u}-z_{r}-l_{2})+m_{u}g_{m}=0 \end{array}\right.$$

where $m_{s}$ denotes the sprung mass, $m_{u}$ denotes the unsprung mass, $z_{s}$ is the displacement of the sprung mass in the parallel configuration, $z_{u}$ denotes the displacement of the unsprung mass in the parallel configuration, $z_{r}$ represents the displacement of the lunar surface excitation input, k signifies the spring stiffness, c is the damping coefficient, b is the inertance coefficient, $k_{t}$ is the corrected equivalent vertical stiffness of the elastic wheel–regolith system, and $g_{m}$ is the lunar gravitational acceleration.

The suspension static deflection $l_{1}$ and the static compression $l_{2}$ of the equivalent wheel–regolith system can be expressed as

(11) $$\left\{\begin{array}{l} l_{1}=\frac{m_{s}g_{m}}{k}\\ l_{2}=\frac{(m_{s}+m_{u})g_{m}}{k_{t}} \end{array}\right.$$

The term $k_{t}(z_{u}-z_{r}-l_{2})$ presents the equivalent normal supporting force generated by the elastic wheel–regolith interaction. This support force exists as long as the wheel remains in contact with the lunar surface; when the wheel loses contact with the lunar surface, this term becomes zero. Therefore, before establishing the corresponding dynamic equations, the contact state between the wheel and the ground must first be determined.

The relative wheel–ground displacement is defined as $z_{t}=z_{u}-z_{r}$. When the relative wheel–ground displacement $z_{t}$ is less than the static compression $l_{2}$ of the equivalent wheel–regolith system, it indicates that the lunar rover’s wheel is in contact with the lunar soil. Conversely, when the relative wheel–ground displacement $z_{t}$ exceeds the static compression $l_{2}$, it indicates that the lunar rover’s wheel has separated from the lunar soil, which results in wheel lift-off.

Under the present coordinate convention, the wheel–ground contact and separation conditions are expressed as

(12) $$\left\{\begin{array}{l} z_{t}=z_{u}-z_{r}<l_{2}\;\;\;\;\;\;\mathrm{contact}\;\mathrm{state}\\ z_{t}=z_{u}-z_{r}\geq l_{2}\;\;\;\;\;\;\mathrm{separation}\;\mathrm{state} \end{array}\right.$$

When the wheel is in contact with the lunar surface, the static terms in Equation (11) are balanced by $l_{1}$ and $l_{2}$. Thus, the contact-state dynamic equations of the parallel inerter–damper suspension can be written as

(13) $$\left\{\begin{array}{l} m_{s}{\ddot{z}}_{s}+k(z_{s}-z_{u})+c({\dot{z}}_{s}-{\dot{z}}_{u})+b({\ddot{z}}_{s}-{\ddot{z}}_{u})=0\\ m_{u}{\ddot{z}}_{u}-k(z_{s}-z_{u})-c({\dot{z}}_{s}-{\dot{z}}_{u})-b({\ddot{z}}_{s}-{\ddot{z}}_{u})+k_{t}(z_{u}-z_{r})=0 \end{array}\right.\;\;\;(z_{u}-z_{r}<l_{2})$$

When the wheel separates from the lunar surface, the equivalent wheel–regolith supporting force is removed. The separation-state dynamic equations are therefore given by

(14) $$\left\{\begin{array}{l} m_{s}{\ddot{z}}_{s}+k(z_{s}-z_{u}-l_{1})+c({\dot{z}}_{s}-{\dot{z}}_{u})+b({\ddot{z}}_{s}-{\ddot{z}}_{u})+m_{s}g_{m}=0\\ m_{u}{\ddot{z}}_{u}-k(z_{s}-z_{u}-l_{1})-c({\dot{z}}_{s}-{\dot{z}}_{u})-b({\ddot{z}}_{s}-{\ddot{z}}_{u})+m_{u}g_{m}=0 \end{array}\right.\;\;\;(z_{u}-z_{r}\geq l_{2})$$

To compare the effects of different configurations of inerter–damper connections, a suspension system with an inerter–damper series configuration was further considered. In this configuration, the inerter element and damper are connected in series, and a virtual displacement $z_{bs}$ is introduced to describe the internal motion of the series branch. The force transmitted through the series inerter–damper branch is denoted as $u_{s}$. Considering the static deflection of the suspension and the static compression of the equivalent wheel–regolith system, the general governing equations can be written as

(15) $$\left\{\begin{array}{l} m_{s}{\ddot{z}}_{ss}+k(z_{ss}-z_{us}-l_{1})+u_{s}+m_{s}g_{m}=0\\ m_{u}{\ddot{z}}_{us}+k_{t}(z_{us}-z_{rs}-l_{2})-k(z_{ss}-z_{us}-l_{1})-u_{s}+m_{u}g_{m}=0\\ u_{s}=b({\ddot{z}}_{ss}-{\ddot{z}}_{bs})=c({\dot{z}}_{bs}-{\dot{z}}_{us})\; \end{array}\right.\;$$

where $z_{ss}$, $z_{us}$, $z_{bs}$ and $z_{rs}$ are the sprung-mass displacement, unsprung-mass displacement, virtual displacement, and lunar-surface excitation of the series configuration, respectively.

During wheel–ground contact, the control equations are expressed as

(16) $$\left\{\begin{array}{l} m_{s}{\ddot{z}}_{ss}+k(z_{ss}-z_{us})+u_{s}=0\\ m_{u}{\ddot{z}}_{us}+k_{t}(z_{us}-z_{rs})-k(z_{ss}-z_{us})-u_{s}=0\\ u_{s}=b({\ddot{z}}_{ss}-{\ddot{z}}_{bs})=c({\dot{z}}_{bs}-{\dot{z}}_{us})\; \end{array}\right.\;\;\;\;(z_{us}-z_{rs}<l_{2})$$

When the wheel is separated from the lunar surface, the wheel–regolith supporting force is removed, and the corresponding equations are expressed as

(17) $$\left\{\begin{array}{l} m_{s}{\ddot{z}}_{ss}+k(z_{ss}-z_{us}-l_{1})+u_{s}+m_{s}g_{m}=0\\ m_{u}{\ddot{z}}_{us}-k(z_{ss}-z_{us}-l_{1})-u_{s}+m_{u}g_{m}=0\\ u_{s}=b({\ddot{z}}_{ss}-{\ddot{z}}_{bs})=c({\dot{z}}_{bs}-{\dot{z}}_{us}) \end{array}\right.\;\;\;\;(z_{us}-z_{rs}\geq l_{2})$$

For comparison, the conventional suspension considered in the subsequent analysis is an independently defined passive spring-damper suspension without an inerter element. It serves as the baseline reference configuration for evaluating the influence of the parallel and series inerter–damper suspensions.

### 2.3. Non-Dimensionalization of the Dynamic Model

The static compression $l_{2}$ of the equivalent wheel–regolith system is taken as the characteristic length L:

(18) $$L=l_{2}=\frac{(m_{s}+m_{u})g}{k_{t}}$$

The characteristic length depends on gravitational acceleration. For the controlled Earth–Moon comparison, the masses and equivalent vertical stiffness are held constant. Consequently, the same dimensional road-displacement amplitude corresponds to different dimensionless amplitudes under Earth and lunar gravity.

For non-dimensionalization, the characteristic time is defined by:

(19) $$\tau =\omega_{A}t\;,\;\omega_{A}=\sqrt{\frac{k}{m_{s}}}$$

The following dimensionless variables and parameters are introduced:

(20) $$\;\mathrm{Parallel}\;Z_{s}=\frac{z_{s}}{L}\;,\;Z_{u}=\frac{z_{u}}{L}\;,\;Z_{r}=\frac{z_{r}}{L}$$

(21) $$\mathrm{Series}\;Z_{ss}=\frac{z_{ss}}{L}\;,\;Z_{us}=\frac{z_{us}}{L}\;,\;Z_{rs}=\frac{z_{rs}}{L},Z_{bs}=\frac{z_{bs}}{L}$$

(22) $$\xi =\frac{c}{2m_{s}\omega_{A}}=\frac{c}{2\sqrt{km_{s}}}\;,\;\beta =\frac{b}{m_{s}}\;,\;\varepsilon =\frac{m_{u}}{m_{s}}\;,\;\alpha =\frac{k_{t}}{k}$$

where $Z_{s}$, $Z_{u}$ and $Z_{r}$ are the variables obtained from $z_{s}$, $z_{u}$ and $z_{r}$ after non-dimensionalization, $Z_{ss}$, $Z_{us}$, $Z_{rs}$ and $Z_{bs}$ are the variables obtained from $z_{ss}$, $z_{us}$, $z_{rs}$ and $z_{bs}$ after non-dimensionalization, ξ is the damper ratio, β is the inerter-to-mass ratio, ε is the mass ratio, and α is the stiffness ratio.

The wheel–ground contact and separation in dimensionless form can be expressed as

(23) $$\left\{\begin{array}{l} Z_{u}-Z_{r}<1\;\;\;\;\;\;\mathrm{contact}\;\mathrm{state}\\ Z_{u}-Z_{r}\geq 1\;\;\;\;\;\;\mathrm{separation}\;\mathrm{state} \end{array}\right.$$

(24) $$\left\{\begin{array}{l} Z_{us}-Z_{rs}<1\;\;\;\;\;\;\mathrm{contact}\;\mathrm{state}\\ Z_{us}-Z_{rs}\geq 1\;\;\;\;\;\;\mathrm{separation}\;\mathrm{state} \end{array}\right.$$

Therefore, the non-dimensional equations of the contact state for the inerter–damper parallel configuration of the suspension system can be expressed as

(25) $$\left\{\begin{array}{l} {\ddot{Z}}_{s}+(Z_{s}-Z_{u})+2\xi ({\dot{Z}}_{s}-{\dot{Z}}_{u})+\beta ({\ddot{Z}}_{s}-{\ddot{Z}}_{u})=0\\ \varepsilon {\ddot{Z}}_{u}-(Z_{s}-Z_{u})-2\xi ({\dot{Z}}_{s}-{\dot{Z}}_{u})-\beta ({\ddot{Z}}_{s}-{\ddot{Z}}_{u})+\alpha (Z_{u}-Z_{r})=0 \end{array}\right.\;\;\;\;{\;(Z}_{u}-Z_{r}<1)$$

In the separation state, the wheel–regolith supporting term is no longer active, and the dimensionless equations can be expressed as

(26) $$\left\{\begin{array}{l} {\ddot{Z}}_{s}+(Z_{s}-Z_{u})+2\xi ({\dot{Z}}_{s}-{\dot{Z}}_{u})+\beta ({\ddot{Z}}_{s}-{\ddot{Z}}_{u})=0\\ \varepsilon {\ddot{Z}}_{u}-(Z_{s}-Z_{u})-2\xi ({\dot{Z}}_{s}-{\dot{Z}}_{u})-\beta ({\ddot{Z}}_{s}-{\ddot{Z}}_{u})+\alpha =0 \end{array}\right.\;\;\;\;{\;(Z}_{u}-Z_{r}\geq 1)$$

Using the same dimensionless parameters, the contact-state equations of the series inerter–damper suspension can be rewritten as

(27) $$\left\{\begin{array}{l} {\ddot{Z}}_{ss}+\left(Z_{ss}-Z_{us}\right)+F_{s}=0\;\\ \varepsilon {\ddot{Z}}_{us}+\alpha \left(Z_{us}-Z_{rs}\right)-\left(Z_{ss}-Z_{us}\right)-F_{s}=0\\ F_{s}=\beta \left({\ddot{Z}}_{ss}-{\ddot{Z}}_{bs}\right)\\ F_{s}=2\xi \left({\dot{Z}}_{bs}-{\dot{Z}}_{us}\right) \end{array}\right.\;\;\;\;(Z_{us}-Z_{rs}<1)$$

where $F_{s}=(u_{s}/kL)$ is the dimensionless force transmitted by the series inerter–damper branch.

In the separation state, the dimensionless equations can be expressed as

(28) $$\left\{\begin{array}{l} {\ddot{Z}}_{ss}+\left(Z_{ss}-Z_{us}\right)+F_{s}=0\;\\ \varepsilon {\ddot{Z}}_{us}-\left(Z_{ss}-Z_{us}\right)-F_{s}+\alpha =0\\ F_{s}=\beta \left({\ddot{Z}}_{ss}-{\ddot{Z}}_{bs}\right)\\ F_{s}=2\xi \left({\dot{Z}}_{bs}-{\dot{Z}}_{us}\right) \end{array}\right.\;\;\;\;(Z_{us}-Z_{rs}\geq 1)$$

To analyze the steady-state response characteristics of the system, it is assumed that the wheels are subjected to harmonic road excitation. 

The road excitation is taken as a cosine function

(29) $$Z_{r}=Y_{0}\cos\left(\gamma \tau \right)Y_{0}=\frac{y_{0}}{L}$$

where $\gamma =(\omega /\omega_{A})$ is the non-dimensional frequency ratio, ω is the circular frequency of the road excitation, $y_{0}$ is the dimensional amplitude of the harmonic road displacement, expressed in meters, and $Y_{0}=y_{0}/L$ is its dimensionless counterpart. The same value of $y_{0}$ is used for both gravity conditions. Based on Equations (25)–(28), the dynamic responses of the parallel and series inerter–damper suspensions can be solved and compared under the same wheel–regolith equivalent stiffness and lunar-surface excitation.

For unified notation, the subscript i=p denotes the parallel inerter–damper suspension, while i=s denotes the series inerter–damper suspension. Under the harmonic lunar-surface excitation, the steady-state responses of the sprung mass and unsprung mass are assumed as

(30) $$Z_{s,i}=A_{1,i}\sin(\gamma \tau )+B_{1,i}\cos(\gamma \tau )$$

(31) $$Z_{u,i}=A_{2,i}\sin(\gamma \tau )+B_{2,i}\cos(\gamma \tau )$$

The unknown coefficients $A_{1,i}$, $B_{1,i}$, $A_{2,i}$, and $B_{2,i}$ are obtained by substituting the harmonic response forms into the corresponding nondimensional governing equations. The detailed algebraic derivation is given in Appendix A.

The wheel–ground relative displacement is defined as

(32) $$Z_{t,i}=Z_{u,i}-Z_{r}$$

Substituting Equations (29) and (31) gives

(33) $$Z_{t,i}=A_{2,i}\sin(\gamma \tau )+(B_{2,i}-Y_{0})\cos(\gamma \tau )$$

Accordingly, the amplitudes of the sprung-mass displacement, suspension dynamic travel, and wheel–ground relative displacement are respectively expressed as

(34) $$\left|Z_{s,i}\right|=\sqrt{{A_{1,i}}^{2}+{B_{1,i}}^{2}}$$

(35) $$\left|Z_{s,i}-Z_{u,i}\right|=\sqrt{{\left(A_{1,i}-A_{2,i}\right)}^{2}+{\left(B_{1,i}-B_{2,i}\right)}^{2}}$$

(36) $$\left|Z_{t,i}\right|=\sqrt{{A_{2,i}}^{2}+{\left(B_{2,i}-Y_{0}\right)}^{2}}$$

## 3. Wheel Lift-Off Response and Boundary Analysis

### 3.1. Parameter Settings

This section is based on the dimensionless piecewise quarter-vehicle model established in Section 2. Therefore, to conduct a tractable theoretical analysis and to investigate the influence of lunar gravity and inerter–damper configurations on wheel–ground separation, a set of representative dimensionless parameters is assumed in this section. These parameters are used as baseline values for comparative analysis rather than as final design values. Unless otherwise specified, the suspension parameters are kept constant, while the inertance ratio is varied to evaluate its effect on the wheel–ground separation boundary and dynamic response. The adopted dimensionless parameters are listed in Table 2.

### 3.2. Numerical Verification

The numerical implementation of the model was examined through response comparisons across excitation amplitudes, following the multi-condition comparison approach used in lunar-rover suspension studies [42]. Two formulations of the present model were considered: a direct-equation formulation integrated using an adaptive Runge–Kutta method and a mechanical-network formulation integrated using the average-acceleration Newmark method. Newmark-based time stepping has also been applied to lunar-rover contact-separation dynamics [41]. For the series configuration, the two formulations used the branch force and the internal-node coordinate, respectively, while retaining the same unilateral wheel–ground contact law.

The verification cases used ε = 0.1, ξ = 0.3856, α = 6.4, and γ = 1.5. The conventional suspension, parallel configuration with β = 0.2, and series configuration with β = 0.8 were subjected to the harmonic input $Z_{r}=Y\cos(\gamma \tau )$, with Y = 3.7117 and Y = 7.4234. The enhanced amplitude produced contact-separation transitions in all three configurations. Displacements were measured relative to the flat-road static equilibrium, and the initial displacement deviations, velocities, and series branch force were set to zero.

The adaptive Runge–Kutta calculations used relative and absolute tolerances of $10^{-9}$ and $10^{-11}$, respectively, and a maximum time step of 0.01. Newmark time steps of h = 0.008, 0.004, and 0.002 were examined. Comparisons were performed at common time points over τ = 20–60. The NRMSE was calculated by normalizing the root-mean-square difference between the two responses by the range of the direct-equation response within the same interval.

For a response variable x, the range-normalized root-mean-square error was defined as

(37) $$E_{x}=\frac{\sqrt{\frac{1}{N}\sum_{i=1}^{N}{\left({x_{i}}^{MN}-{x_{i}}^{DE}\right)}^{2}}}{\max{x_{i}}^{DE}-\min{x_{i}}^{DE}}\times 100\%$$

Here, DE and MN denote the direct-equation and mechanical-network implementations, respectively, and N is the number of comparison samples. The normalization range was calculated separately for each response and verification case.

**Figure 4:**
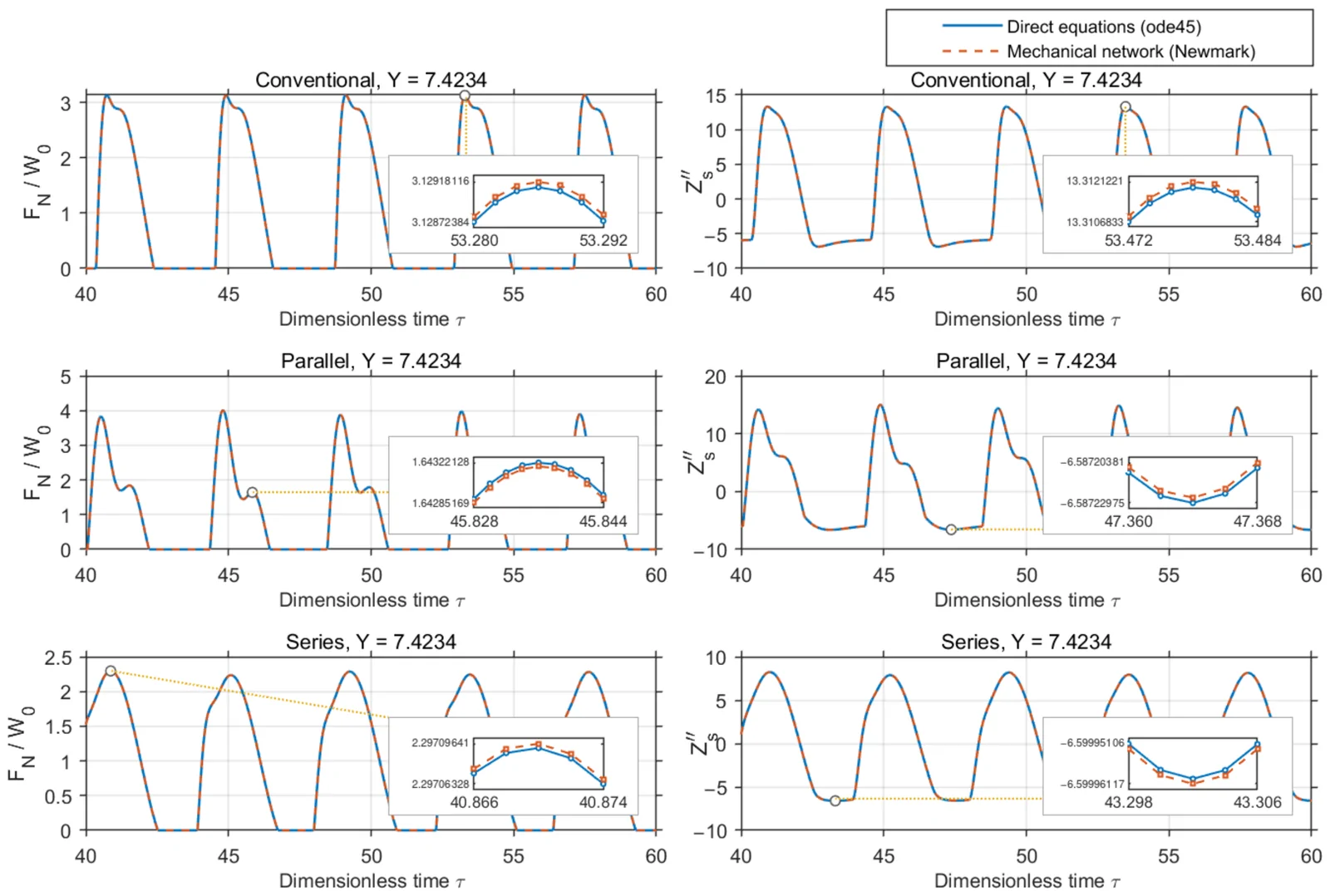
Comparison of normalized wheel–ground normal force and dimensionless sprung acceleration obtained using two numerical implementations under harmonic excitation with Y = 7.4234.

Figure 4 compares the normalized wheel–ground normal force and dimensionless sprung-mass acceleration obtained from the two numerical implementations at Y = 7.4234 over τ = 40–60, where the normal force is normalized by the static wheel load $W_{0}$. All three suspension configurations exhibit alternating contact and separation, as indicated by the positive-force portions and zero-force intervals. The two implementations give closely matching force peaks, separation intervals, and acceleration waveforms. The agreement therefore extends across successive contact-separation transitions and the associated sprung-mass response.

Table 3 summarizes the response errors and off-ground time fractions for the six verification cases over τ=20−60, with a Newmark time step of h = 0.002. The maximum acceleration and normal-force NRMSE values are 0.00314% and 0.004189%, respectively, both occurring in the conventional suspension at Y = 7.4234. At Y = 3.7117, both implementations predict continuous contact for the parallel and series configurations; at Y = 7.4234, both predict intermittent separation for all three configurations. At the common sampling resolution, the off-ground time fractions are identical in five cases. The remaining case, corresponding to the series configuration at Y = 7.4234, gives 31.2575% and 31.2625% for the direct-equation and mechanical-network formulations, respectively, differing by 0.005 percentage points.

**Figure 5:**
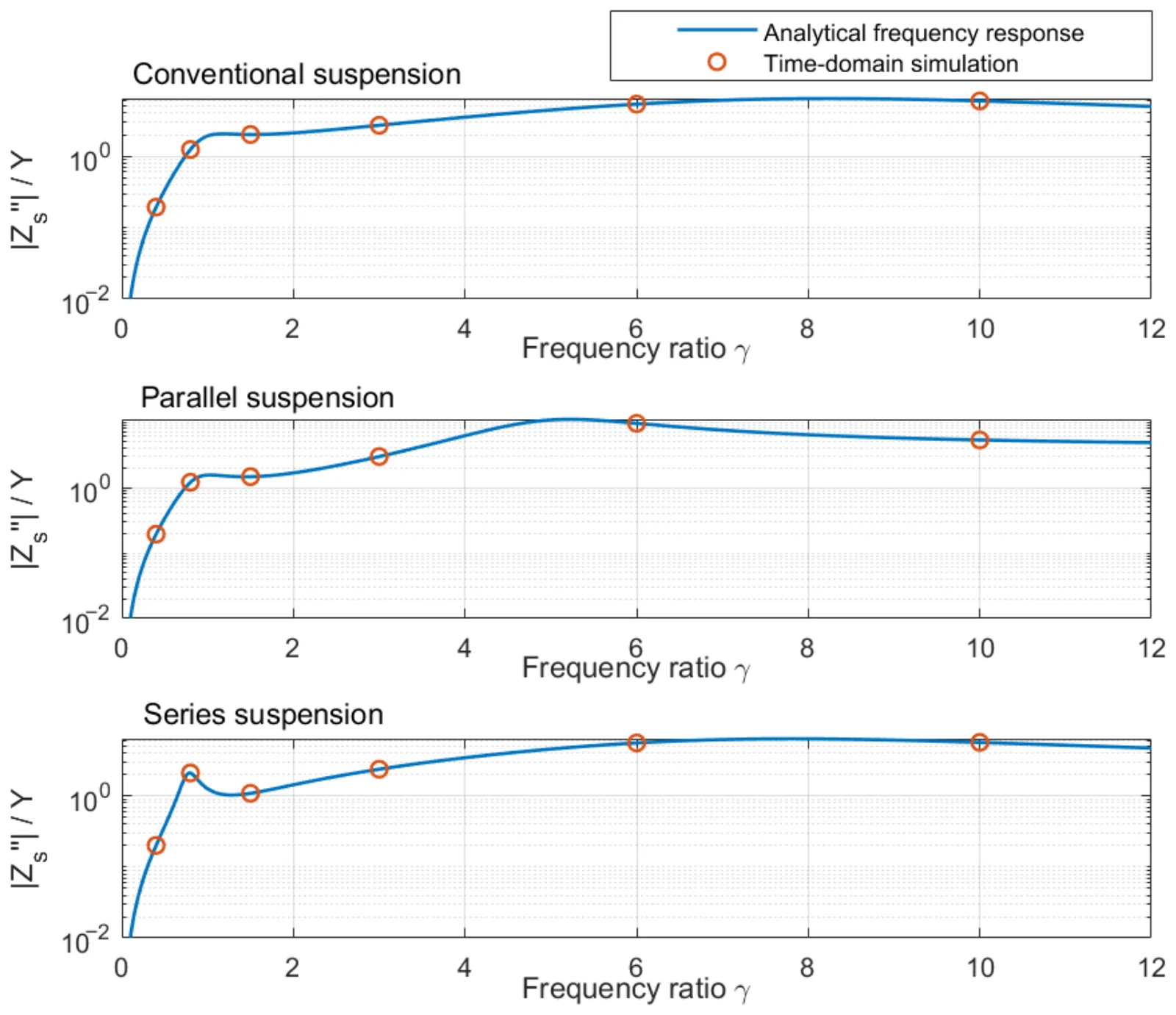
Comparison of analytical and time-domain acceleration gains for three suspension configurations under continuous wheel–ground contact.

Figure 5 compares the analytical acceleration gains with the time-domain results for the three suspension configurations. An excitation amplitude of Y = 0.001 was adopted to maintain continuous wheel–ground contact over the investigated frequency range for comparison with the analytical frequency response. The gain is defined as the ratio of the steady-state dimensionless sprung-mass acceleration amplitude to the excitation amplitude. At γ=0.4, 0.8, 1.5, 3, 6 and 10, the time-domain results closely match the corresponding analytical curves for all three configurations, indicating consistent predictions of the acceleration gain across the selected frequencies under continuous contact.

The agreement between the two numerical implementations, together with the time-step refinement results, supports the consistency and convergence of the computed responses.

### 3.3. Necessity of Considering Wheel Lift-Off and Frequency-Response Characteristics

**Figure 6:**
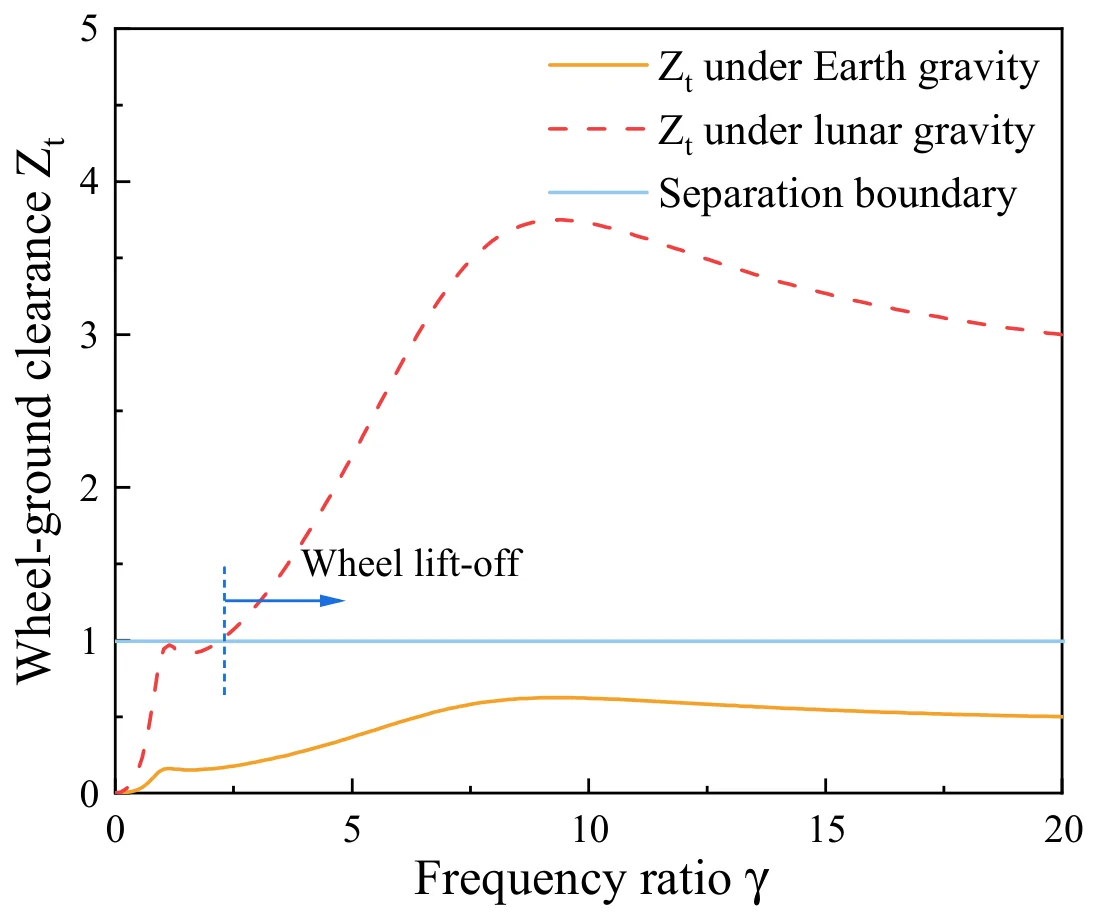
Dimensionless wheel–ground relative displacement under Earth gravity and lunar gravity.

Under lunar gravity, the static normal load of the wheel is significantly reduced. Therefore, the wheel is more likely to lose contact with the ground under vertical excitation. Figure 6 shows the dimensionless wheel–ground relative displacement under Earth gravity and lunar gravity. With the same suspension parameters and dimensional road-displacement amplitude, the wheel remains in contact with the ground under Earth gravity, whereas wheel lift-off occurs under lunar gravity. This indicates that wheel lift-off must be considered in the suspension analysis of lunar rovers.

**Figure 7:**
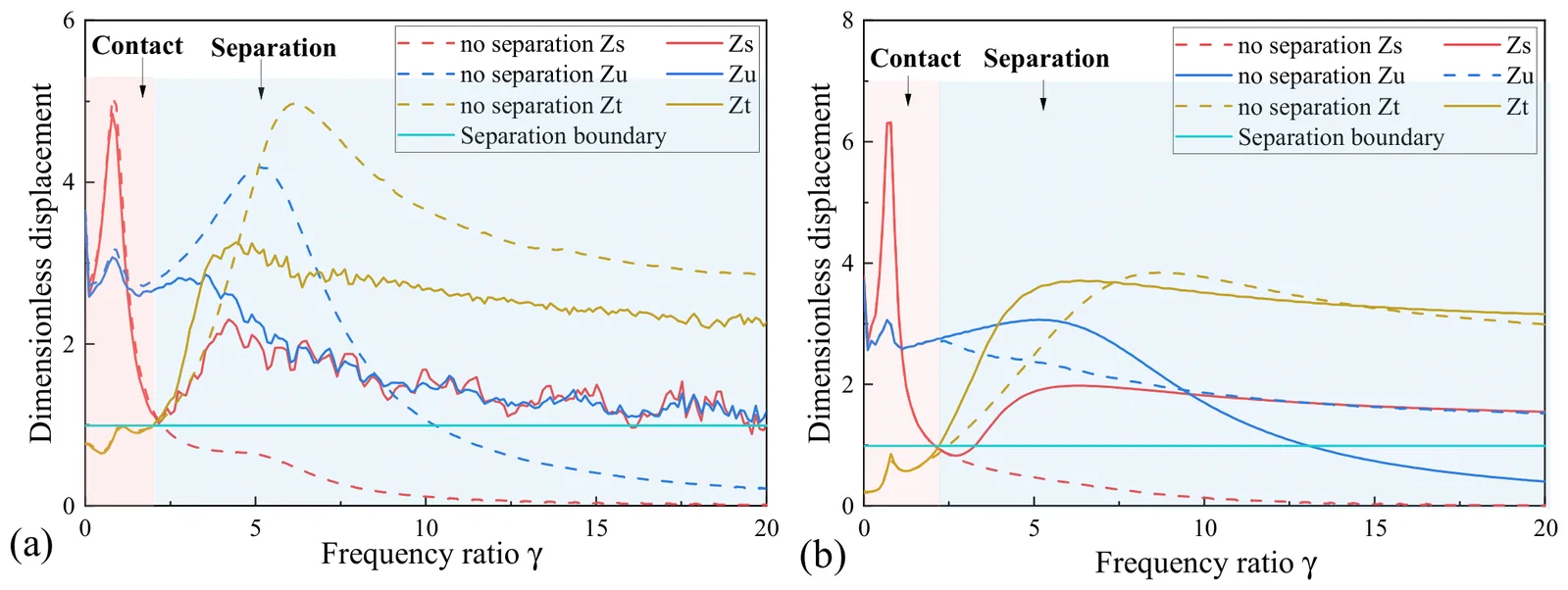
Comparison of dimensionless displacement frequency responses with and without wheel–ground separation for different inerter–damper configurations: (**a**) parallel inerter–damper configuration; (**b**) series inerter–damper configuration.

Figure 7 compares the dimensionless displacement frequency responses of the parallel and series inerter–damper configurations, with and without considering wheel lift-off. The solid lines represent the dimensionless displacement frequency responses when wheel lift-off is considered, whereas the dashed lines represent the responses obtained under the assumption of continuous wheel–ground contact. Before the lift-off frequency is reached, the solid and dashed curves almost coincide. However, after the lift-off frequency, the two responses begin to diverge. When γ=8, the actual dimensionless sprung-mass displacement $Z_{s}$ increases by approximately 80% compared with the case in which wheel lift-off is neglected. If wheel lift-off is ignored, the predicted displacement response will deviate from the actual response, thereby affecting the accuracy of ride comfort evaluation and wheel–ground contact assessment for the manned lunar rover.

Therefore, wheel lift-off must be considered in the dynamic modeling of manned lunar rover suspension systems. Moreover, structural or parameter adjustments should be adopted to reduce the occurrence of wheel lift-off or increase the critical lift-off frequency.

**Figure 8:**
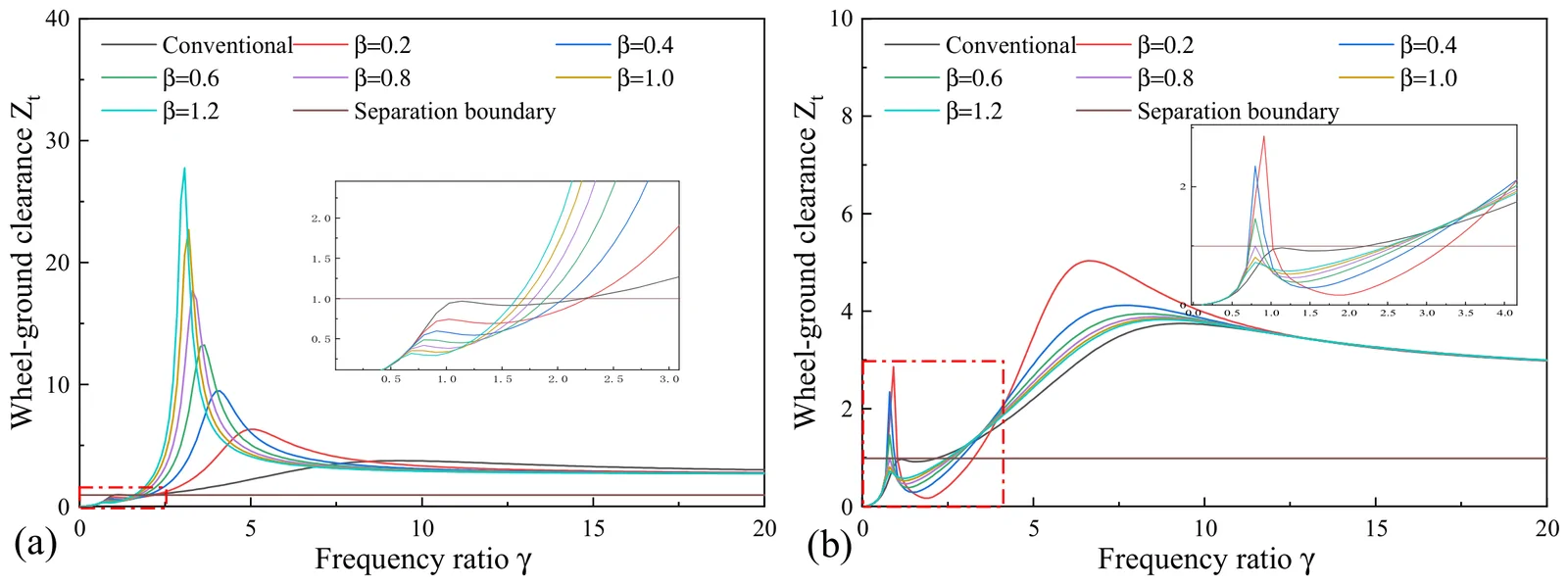
Comparison of the dimensionless wheel–ground relative displacement response between the inerter suspension and the conventional suspension under different inertance ratios: (**a**) parallel inerter–damper configuration; (**b**) series inerter–damper configuration.

Figure 8 shows the effect of different inertance ratios on the dimensionless wheel–ground relative displacement response $Z_{t}$ for the parallel and series inerter–damper configurations. Figure 8a corresponds to the parallel configuration, while Figure 8b corresponds to the series configuration. The horizontal line denotes the wheel–ground separation boundary.

As shown in Figure 8, for the parallel configuration, the inerter provides only a limited increase in the critical frequency ratio of wheel lift-off. Moreover, as the inertance ratio increases, the peak value of $Z_{t}$ in the mid-frequency range increases significantly, which may intensify the wheel–ground clearance response after separation. In contrast, for the series configuration, the $Z_{t}$ curves corresponding to different inertance ratios vary more smoothly, and the intersection with the separation boundary shifts toward a higher frequency range. This indicates that the series configuration has certain advantages in increasing the critical frequency ratio of wheel lift-off. Therefore, from the perspective of wheel–ground contact, the series inerter–damper configuration is more effective in delaying wheel–ground separation.

**Figure 9:**
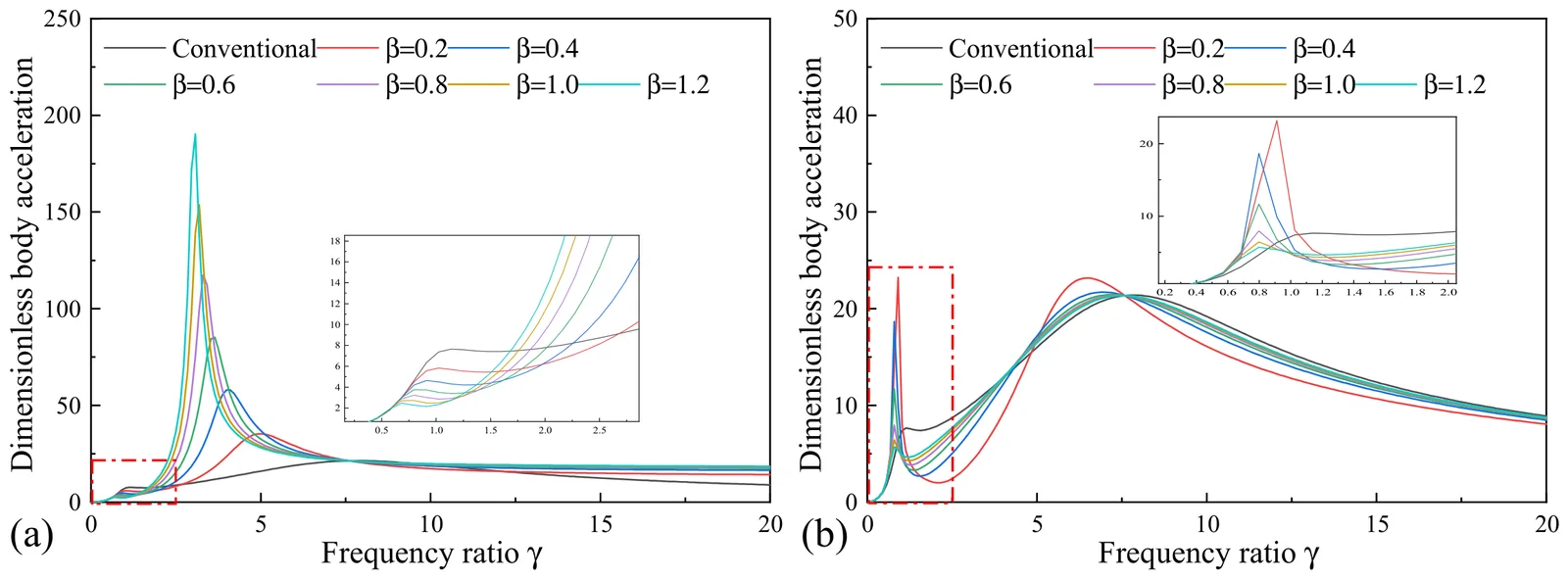
Comparison of dimensionless vehicle body acceleration between the conventional suspension and the inerter suspension under different inertance ratios: (**a**) parallel inerter–damper configuration; (**b**) series inerter–damper configuration.

Figure 9 further compares the dimensionless vehicle body acceleration responses of the conventional suspension and the inerter suspension under different inertance ratios. For the parallel inerter–damper configuration, increasing the inertance ratio can delay wheel lift-off to some extent, but an excessively large inertance ratio also leads to a significant increase in the mid-frequency acceleration peak. Therefore, the case of β = 0.2 provides a reasonable compromise between improving wheel–ground contact and avoiding excessive body acceleration. For the series inerter–damper configuration, the acceleration responses vary more smoothly when β is larger than 0.8, and the wheel–ground clearance response also indicates an improved lift-off boundary. Hence, β = 0.8 is selected as the representative series configuration in the following boundary analysis and time-domain comparison.

### 3.4. Lift-Off Boundary and Numerical Consistency Check

**Figure 10:**
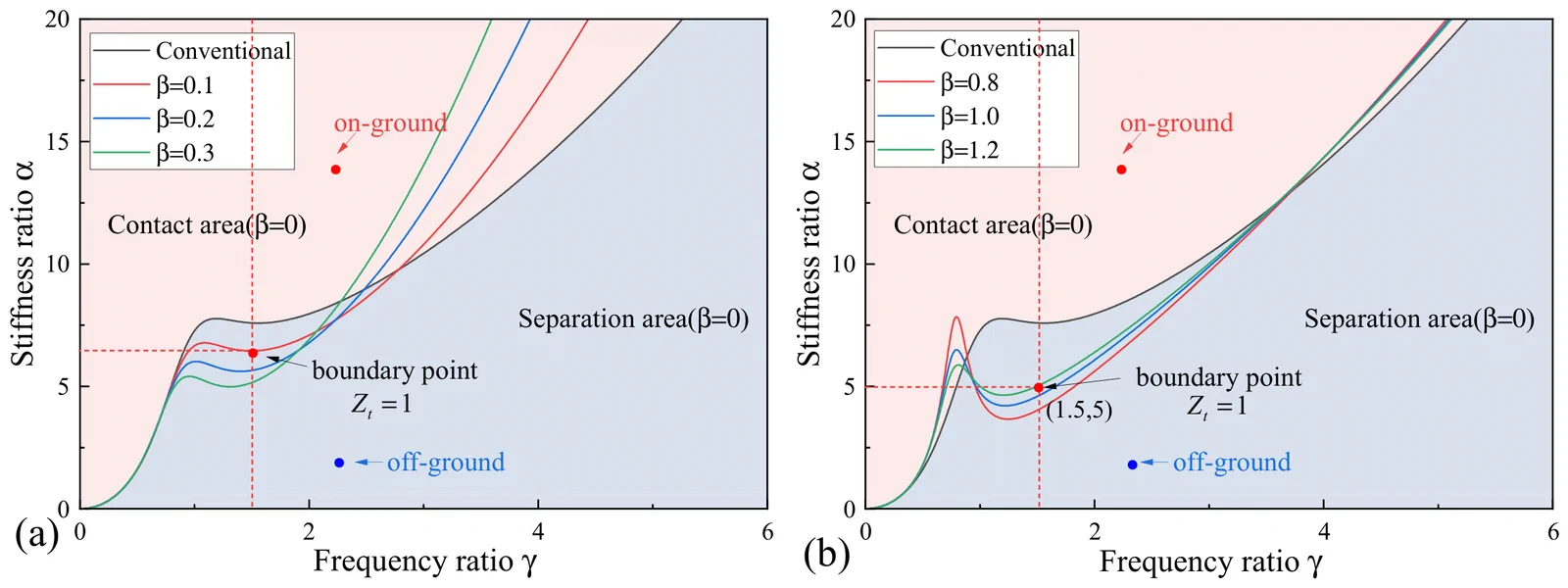
Comparison of wheel–ground separation boundaries between the conventional suspension and the inerter suspension under different inertance ratios: (**a**) parallel inerter–damper configuration; (**b**) series inerter–damper configuration.

The wheel–ground separation boundary is obtained based on the dimensionless wheel–ground clearance response. For each given inertance ratio β, the frequency ratio γ and stiffness ratio α are varied within the prescribed parameter ranges. At each point in the (γ,α) parameter plane, the steady-state dimensionless wheel–ground clearance response $Z_{t}$ is calculated. Wheel lift-off is considered to occur when the maximum value of $Z_{t}$ reaches or exceeds the separation threshold, namely $Z_{t}=1$. Therefore, the points satisfying $Z_{t}=1$ form the wheel–ground separation boundary. The area above the boundary corresponds to continuous wheel–ground contact, whereas the area below the boundary corresponds to wheel lift-off. Based on this criterion, the separation boundaries of the conventional suspension and the inerter suspensions are obtained, as shown in Figure 10.

Figure 10 shows the wheel–ground separation boundaries of the conventional suspension and the inerter suspension under different inertance ratios β. The boundary curves divide the parameter plane into the wheel–ground contact area and the wheel–ground separation area. When the design point is located above the boundary curve, the manned lunar rover remains in contact with the ground; otherwise, wheel–ground separation occurs.

As shown in Figure 10a, for the parallel inerter suspension, the wheel–ground contact area in the low-frequency-ratio range expands significantly as the inertance ratio β increases. The design point P(1.5,6.4) lies in the separation area for the conventional suspension. When β=0.1, it is located on the separation boundary; when β=0.2 and β=0.3, it is in the wheel–ground contact area. This indicates that the parallel inerter–damper configuration can improve the wheel–ground contact state near this design point.

As shown in Figure 10b, the series inerter suspension can also enlarge the wheel–ground contact area. At the design point P(1.5,6.4), the wheel remains in contact with the ground when β=0.8, β=1.0, and β=1.2. Compared with the conventional suspension, both inerter suspensions expand the wheel–ground contact area in the low-frequency-ratio range, which is beneficial for maintaining wheel–ground contact of the manned lunar rover under low-frequency and large-amplitude lunar surface excitations.

**Figure 11:**
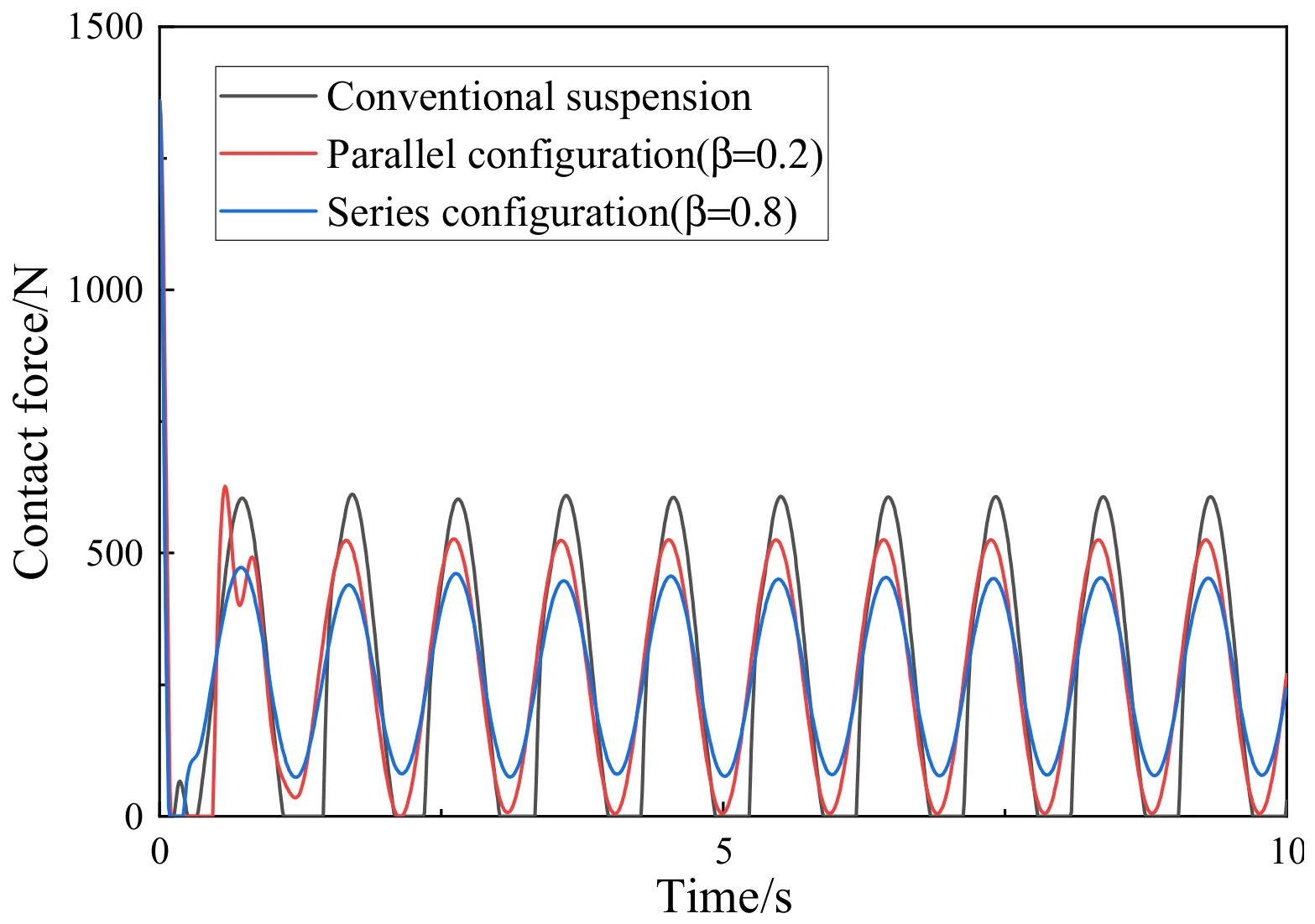
Comparison of wheel–ground contact force between the conventional suspension and the inerter suspension at the design point *P*.

To verify the effectiveness of the wheel–ground separation boundary diagram, the wheel–ground contact force is simulated at the design point P(1.5,6.4), and the results are shown in Figure 11. When the contact force drops to zero, wheel lift-off occurs. It can be observed that the contact force of the conventional suspension drops to zero several times, indicating the occurrence of wheel–ground separation, which is consistent with the separation-region prediction in Figure 10. The series configuration with β=0.8 maintains a positive contact force after the initial transient, while the parallel configuration with β=0.2 approaches zero without the extended zero-force intervals observed for the conventional suspension. At the beginning of the simulation, transient vibration associated with the initial state is superimposed on the response to harmonic excitation, producing additional fluctuations in the contact force. As the transient component decays through damping, the response approaches a repeatable periodic waveform. For the conventional suspension, the zero-force intervals associated with wheel separation also shape the waveform within each cycle. The time-domain contact-force response is consistent with the contact/separation-region prediction in Figure 10, providing a numerical consistency check for the proposed wheel lift-off boundary.

### 3.5. Time-Domain Comparison Under Increased Excitation

To further distinguish the wheel–ground contact characteristics of different suspension configurations, the dimensionless excitation amplitude $Y_{0}$ is increased by one-third so that wheel lift-off becomes more pronounced and the differences among the investigated configurations can be more clearly identified.

**Figure 12:**
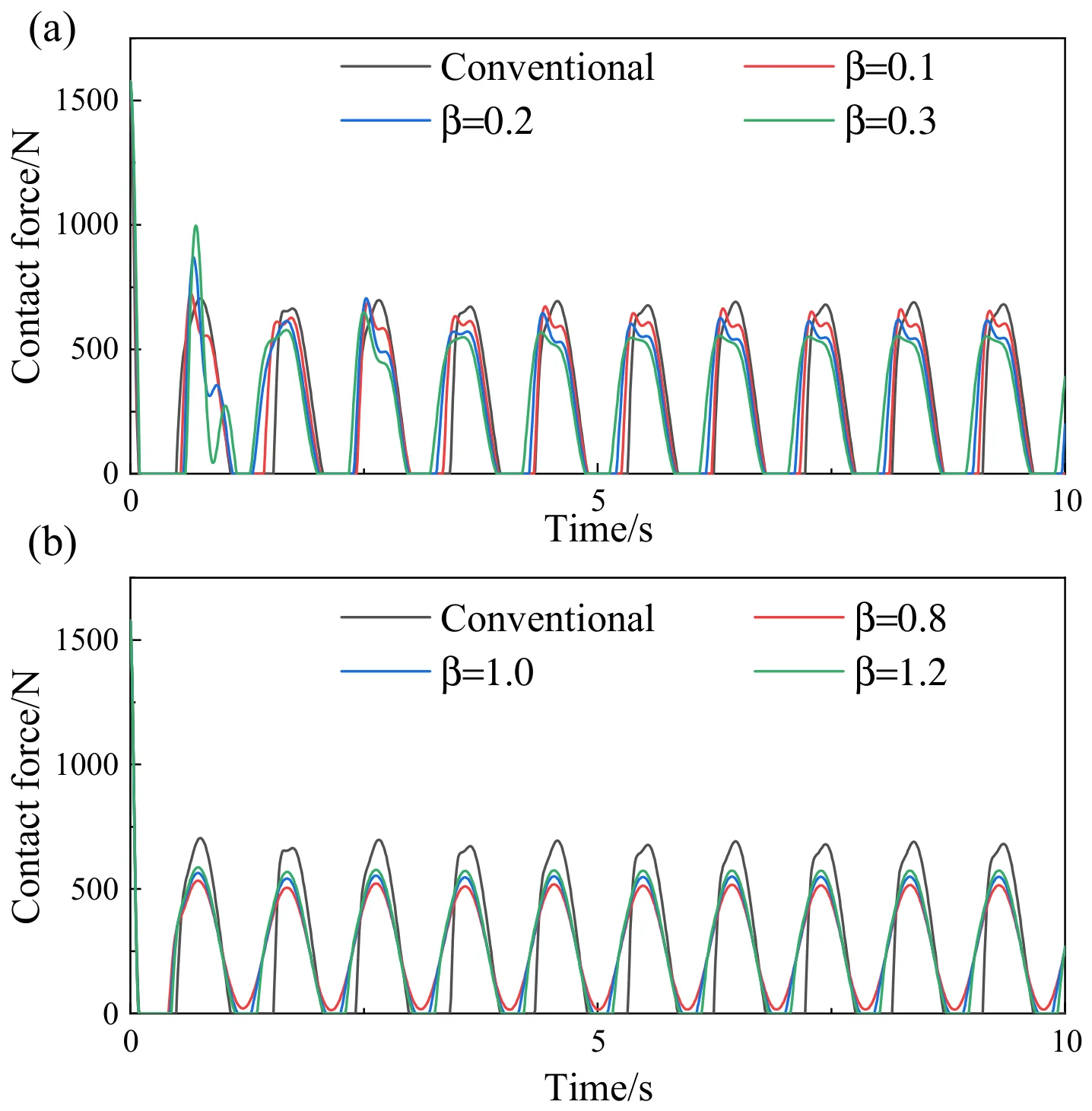
Comparison of wheel–ground contact forces under stronger excitation for different suspension configurations: (**a**) parallel inerter–damper configuration; (**b**) series inerter–damper configuration.

**Figure 13:**
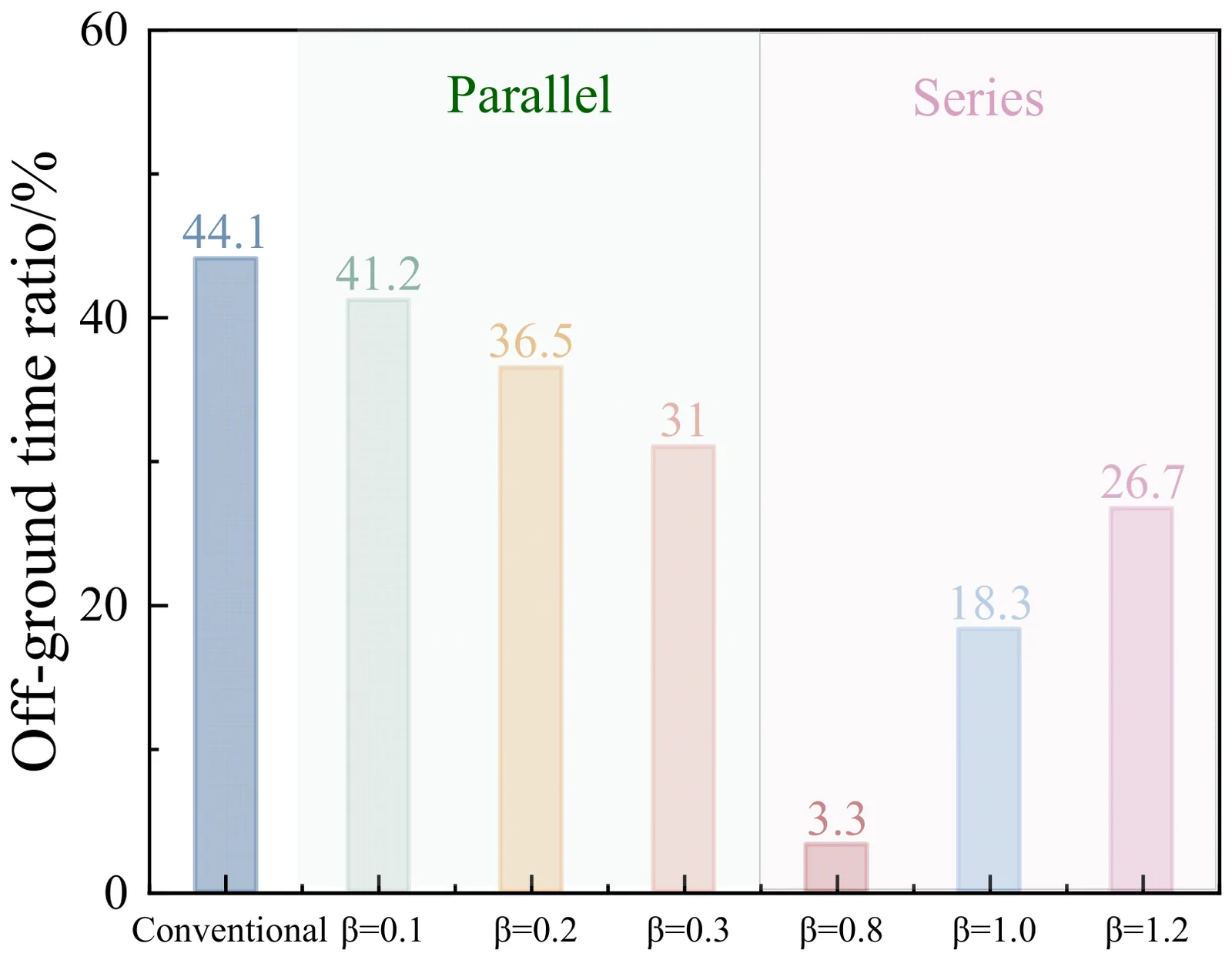
Comparison of the off-ground time ratio for different suspension configurations.

As shown in Figure 12a, compared with the conventional passive suspension, the parallel inerter–damper configuration reduces the peak wheel–ground contact force to a certain extent as the inertance ratio increases. The corresponding off-ground time ratios are shown in Figure 13. For the parallel configuration, the off-ground time ratio decreases from 44.1% for the conventional suspension to 41.2%, 36.5%, and 31.0% when β=0.1, β=0.2 and β=0.3, respectively.

As shown in Figure 12b, the series inerter–damper configuration provides a more significant reduction in wheel lift-off. The off-ground time ratio decreases from 44.1% for the conventional suspension to 3.3% when β=0.8. However, when β further increases to 1.0 and 1.2, the off-ground time ratio increases to 18.3% and 26.7%, respectively. This indicates that the improvement of the series configuration is not monotonic with respect to the inertance ratio, and appropriate parameter selection is required. Among the investigated cases, the series configuration with β=0.8 achieves the lowest off-ground time ratio and shows the best wheel–ground contact performance.

Figure 13 further highlights the non-monotonic dependence of wheel lift-off performance on the inertance ratio. As shown in Figure 13, increasing β from 0.8 to 1.2 increases the off-ground time ratio from 3.3% to 26.7%. Therefore, a larger inertance does not necessarily lead to improved wheel–ground contact retention. The inerter modifies both the effective dynamic impedance and the relative motion between the sprung and unsprung masses, and excessive inertance may consequently shift the dynamic response into an unfavorable region. In the series branch, the inerter and damper transmit the same force, while the relative motion across the branch is shared between them. With damping fixed, changing inertance modifies the amplitude and phase of the transmitted force, thereby affecting the wheel motion relative to the terrain and the duration of contact loss. This result indicates that inertance and damping should be tuned jointly for the excitation range of interest rather than selecting the largest achievable inertance.

Compared to the parallel configuration, the series configuration exhibits a more stable improvement in the separation boundary and a lower wheel off-ground time ratio within the investigated parameter range. Therefore, the series inerter–damper configuration is selected for further parameter optimization.

## 4. Parameter Optimization of the Inerter Suspension for the Manned Lunar Rover

To improve the vibration performance of the inerter suspension system of the manned lunar rover under low-frequency large-amplitude excitation and enhance the lift-off suppression capability of the vehicle, typical low-frequency, large-amplitude excitation models for rough mare and rough upland are established. Subsequently, a multi-objective particle swarm optimization algorithm is employed to obtain the optimal parameters of the suspension system for vibration performance.

### 4.1. Modeling of Low-Frequency Large-Amplitude Excitation on the Lunar Surface

Based on lunar surface probe data from NASA, the roughness coefficient $K_{r}$ of the lunar surface is selected, and the displacement power spectral density (PSD) functions for the excitation of rough mare and rough upland are obtained using curve fitting methods and represented as follows:

(38) $$\left\{\begin{array}{l} S_{q,b}=10^{\left(0.1951{\mathrm{lg}}^{4}X_{b}+2.2864{\mathrm{lg}}^{3}X_{b}+5.0045{\mathrm{lg}}^{2}X_{b}+0.7154\mathrm{lg}X_{b}-3.4239\right)}\\ S_{q,d}=10^{\left(-1.0785{\mathrm{lg}}^{4}X_{d}-3.5729{\mathrm{lg}}^{3}X_{d}-3.5762{\mathrm{lg}}^{2}X_{d}-3.7720\mathrm{lg}X_{d}-4.2427\right)} \end{array}\right.$$

where $S_{q,b}$ and $S_{q,d}$ represent the power spectral density of rough mare and rough upland, and $X_{b}$ and $X_{d}$ represent the spatial frequency of rough mare and rough upland.

Spatial domain road excitations for rough mare and rough upland are generated using the harmonic superposition method. The two types of lunar terrain are defined as follows:

(39) j=b,d

where b represents rough mare, and d represents rough upland.

For the j-th terrain type, the spatial frequency range $\left[n_{\min},n_{\max}\right]$ is divided into N intervals with a constant frequency resolution

(40) $$\Delta n=\frac{n_{\max}-n_{\min}}{N}$$

The center spatial frequency of the i-th interval is expressed as

(41) $$n_{i}=n_{\min}+\left(i-\frac{1}{2}\right)\Delta n\;,\;i=1,2,\cdots ,N$$

According to the PSD function $S_{q,j}(n)$, the amplitude of each harmonic component is given by

(42) $$A_{j,i}=\sqrt{2S_{q,j}(n_{i})\Delta n}$$

Therefore, the continuous lunar terrain excitation in the spatial domain can be written as

(43) $$q_{j}(x)=\sum_{i=1}^{N}\sqrt{2S_{q,j}(n_{i})\Delta n}\sin(2\pi n_{i}x+\theta_{i})$$

where $q_{j}(x)$ denotes the vertical roughness displacement of the j-th lunar terrain, x is the longitudinal traveling distance, $S_{q,j}(n_{i})$ is the displacement PSD at $n_{i}$, and $\theta_{i}$ is a random phase uniformly distributed in [0,2π].

In the numerical implementation, the spatial-frequency range is set to $0.01-0.5\;m^{-1}$ and discretized into 200 harmonic components. A terrain profile length of 200 m with a spatial sampling interval of 0.1 m is adopted. The rover speed is set to 3 m/s. The random phases are generated from a uniform distribution over $\left[0,2\pi \right]$ using a fixed random seed to ensure reproducibility.

**Figure 14:**
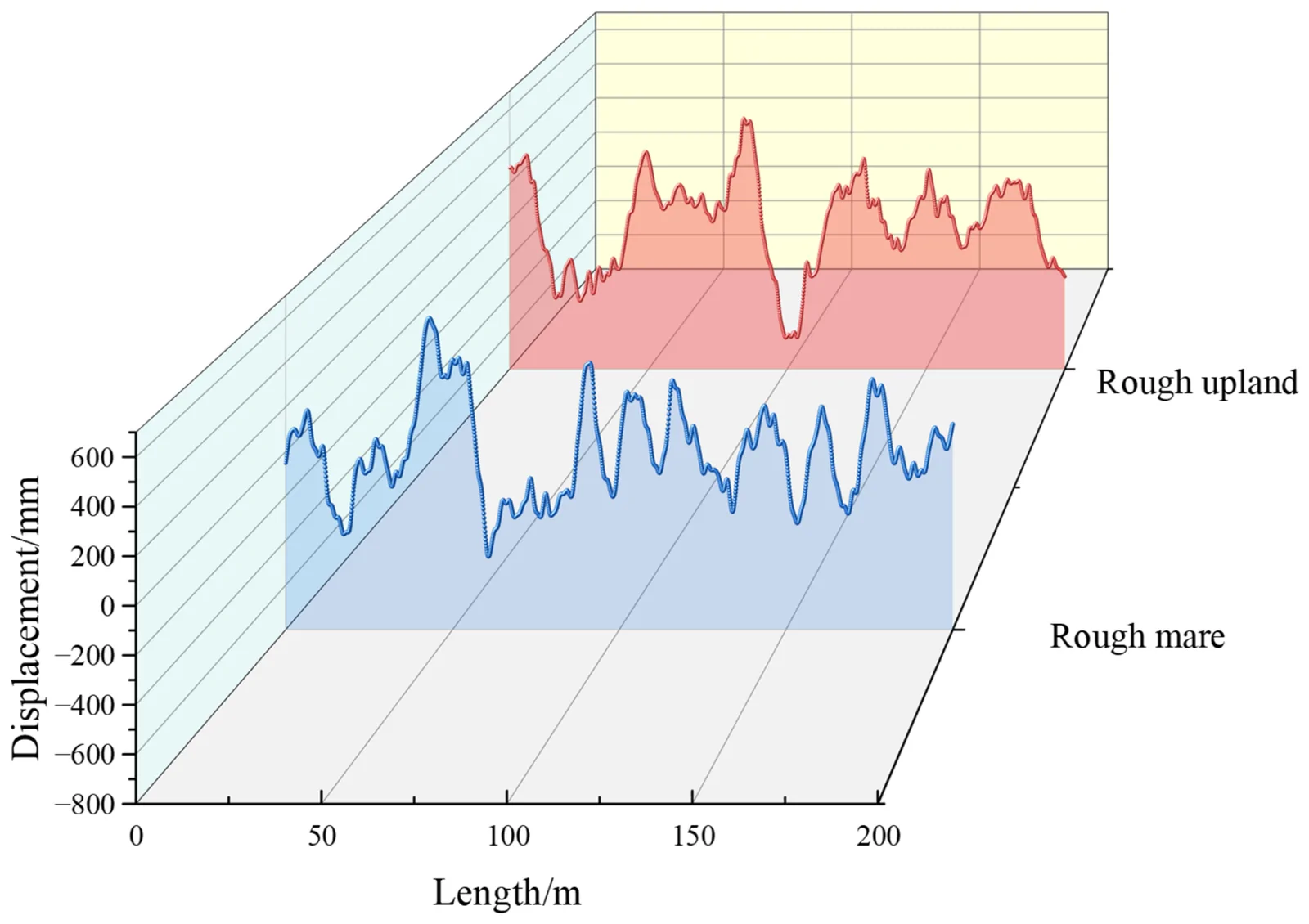
Continuous roughness profiles of rough-mare and rough-upland lunar terrain.

The low-frequency, large-amplitude excitations for rough mare and rough upland are illustrated in Figure 14.

### 4.2. Optimization of Suspension System Parameters

The optimization objective function designed to suppress the vehicle’s lift-off and enhance vibration performance under low-frequency, large-amplitude excitations is as follows:

(44) $$\left\{\begin{array}{l} J_{1}(p)=w_{1}\frac{\sigma_{acc}}{\sigma_{acc}^{ref}}+w_{2}\frac{\sigma_{sws}}{\sigma_{sws}^{ref}}+w_{3}\frac{\sigma_{dtl}}{\sigma_{dtl}^{ref}}\;\mathrm{primary}\;\mathrm{optimization}\;\mathrm{objective}\\ J_{2}(p)=w_{a}\frac{t_{off}}{t_{off}^{ref}}+w_{b}\frac{N}{N^{ref}}\;\mathrm{secondary}\;\mathrm{lift}\text{-}\mathrm{off}\;\mathrm{evaluation}\;\mathrm{objective} \end{array}\right.$$

where $\sigma_{acc}$, $\sigma_{sws}$ and $\sigma_{dtl}$ represent the weighted root mean square values of the vehicle body acceleration, suspension dynamic travel, and tire dynamic load, respectively; $\sigma_{acc}^{ref}$, $\sigma_{sws}^{ref}$ and $\sigma_{dtl}^{ref}$ denote the reference values; $t_{off}$ signifies the time the vehicle is off the ground; N represents the number of times the vehicle is off the ground; and $w_{i},(i=1,2,3,a,b)$ is the weighting coefficient.

Two complementary performance objectives are considered in the parameter-design process. The first objective, $J_{1}$, characterizes the overall vibration performance by combining the normalized RMS values of body acceleration, suspension working space, and dynamic wheel load. The second objective, $J_{2}$, characterizes wheel lift-off performance using the off-ground duration and the number of lift-off events. In the numerical optimization, $J_{1}$ is used as the primary search criterion, while $J_{2}$ is evaluated for the obtained parameter combinations to assess whether the optimized suspension also improves wheel–ground contact performance.

The optimized parameters and search ranges for the inerter suspension system of the manned lunar rover are presented in Table 4.

**Figure 15:**
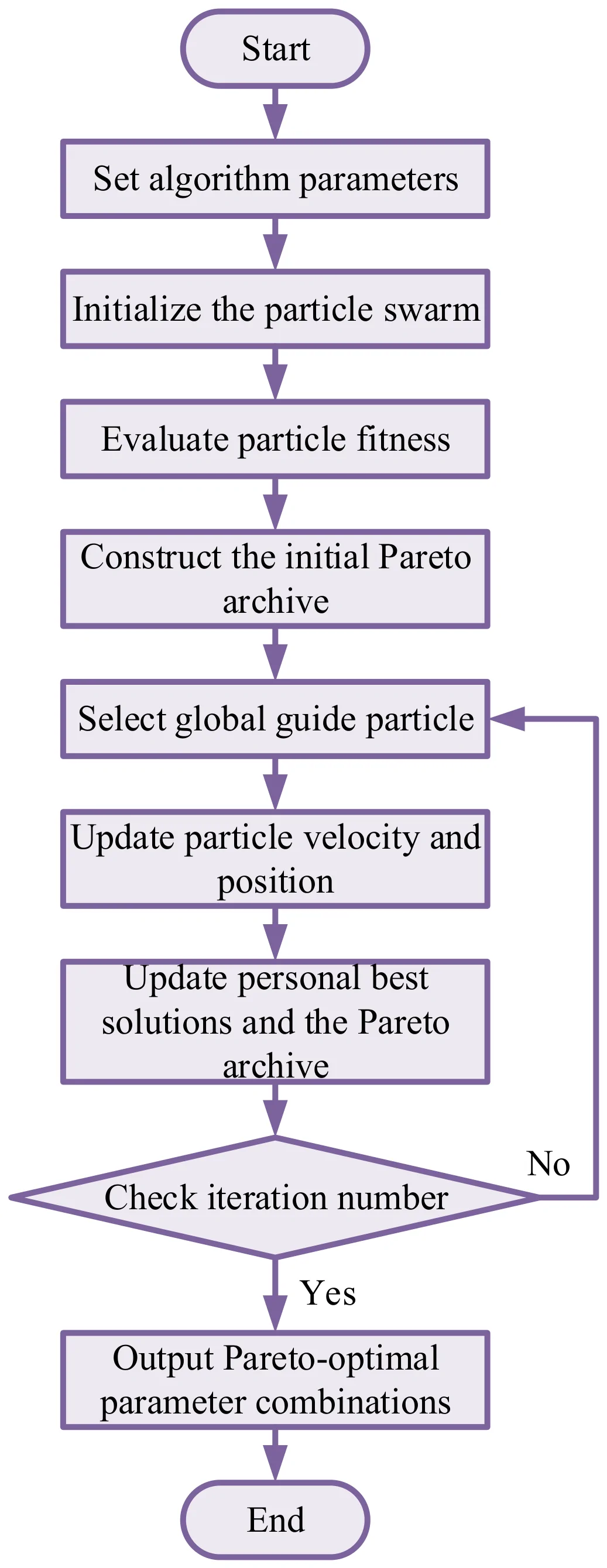
Optimization process of optimization-based parameter design.

The process of obtaining the optimal suspension parameters using a multi-objective particle swarm algorithm is illustrated in Figure 15.

The control parameters of the optimization algorithm encompass population size, dimensionality, and the iteration count required to terminate the optimization process. For the current optimization procedure, the population size is established at 100, the dimensionality is designated as 2, and the termination criterion is specified as 80 iterations. The optimized parameters of the inerter suspension system of the manned lunar rover under rough mare and rough upland excitation inputs are shown in Table 5.

### 4.3. Analysis of Performance Comparison

**Figure 16:**
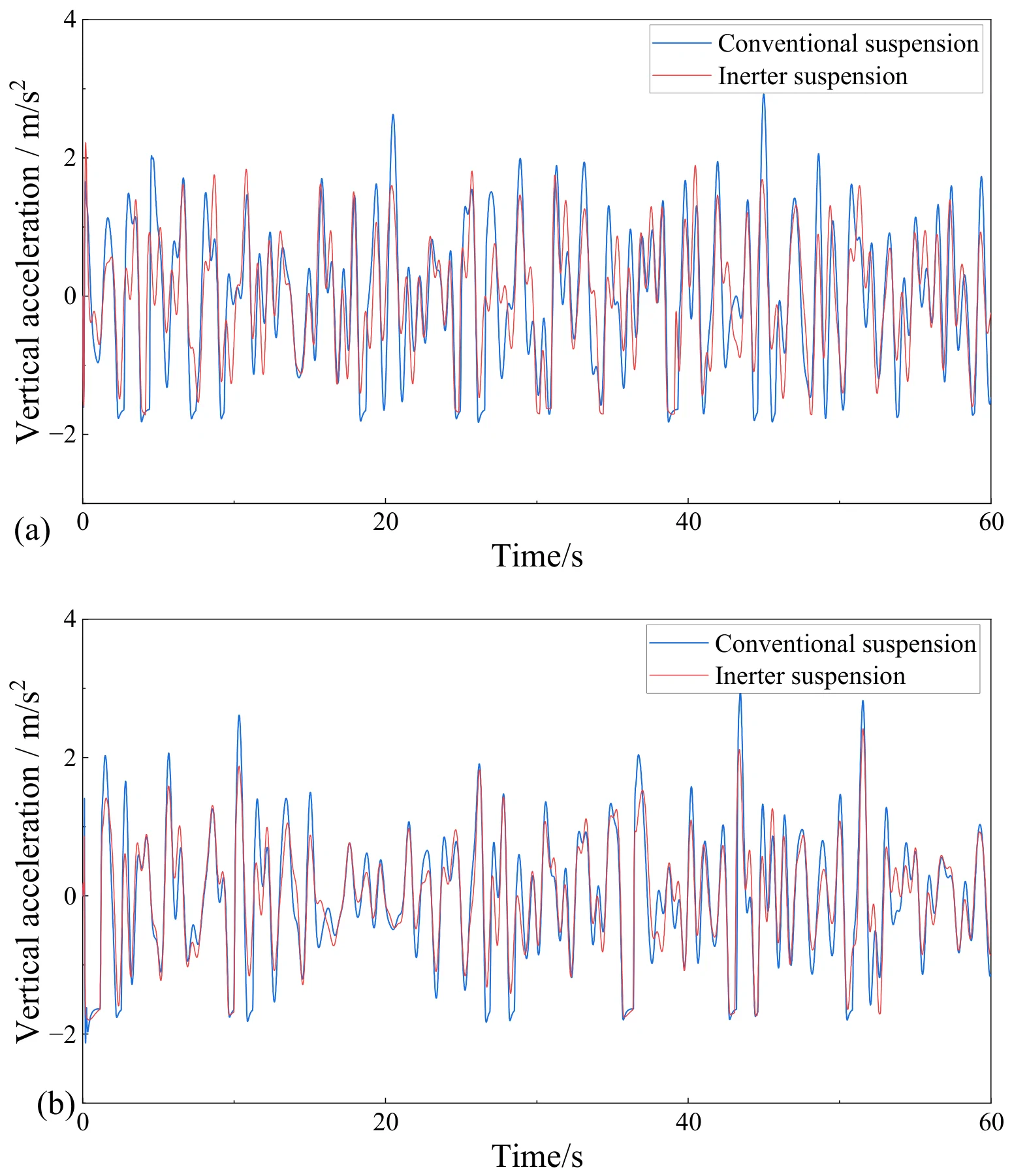
Comparison of vertical acceleration performance: (**a**) rough mare; (**b**) rough upland.

**Figure 17:**
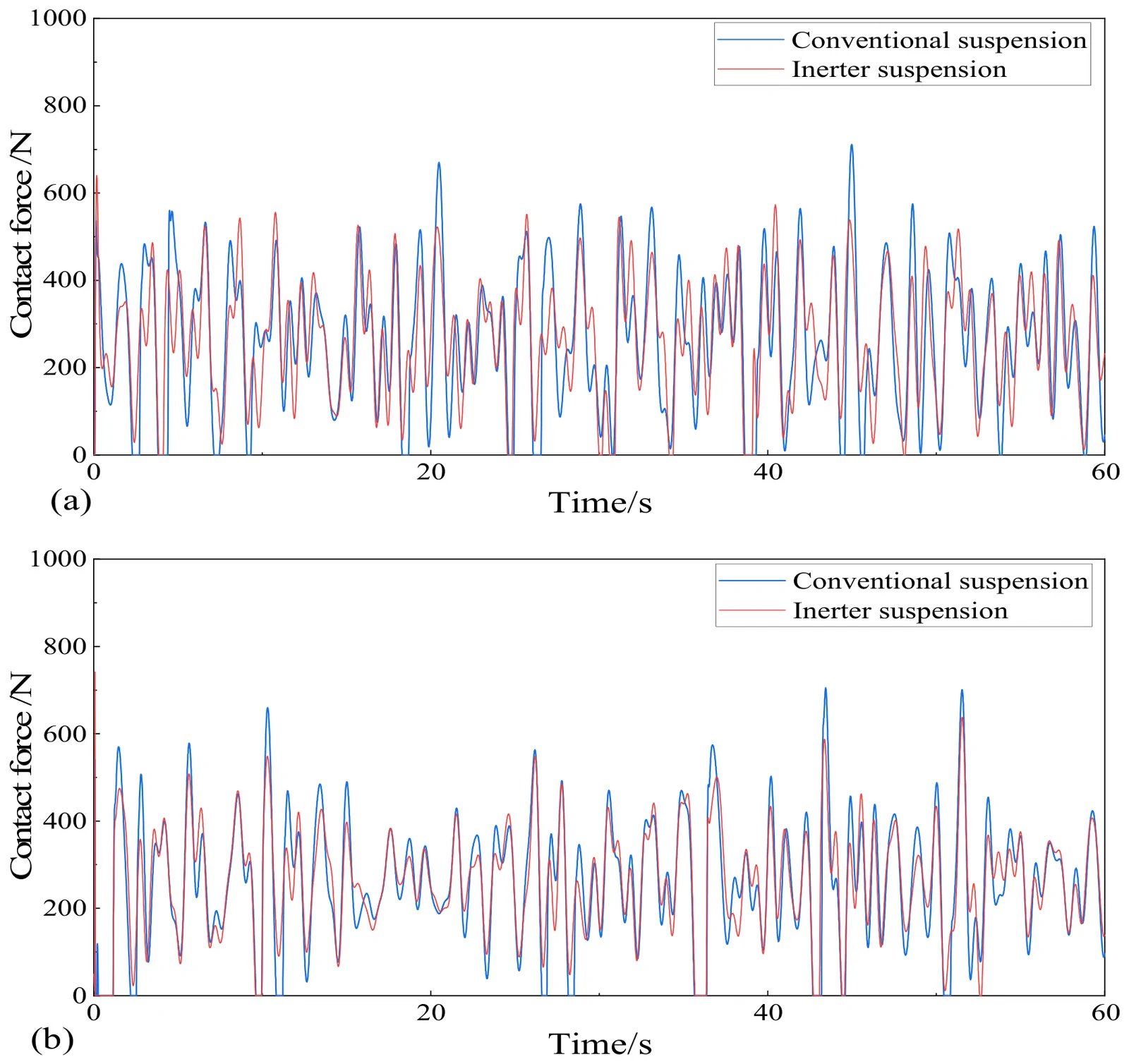
Comparison of wheel–ground contact force performance: (**a**) rough mare; (**b**) rough upland.

Using the optimized parameter combinations in Table 5, the vertical responses of the series inerter suspension are compared with those of the conventional suspension under rough-mare and rough-upland excitations. For each terrain case, both suspensions are subjected to the same terrain excitation history, and their performance metrics are evaluated over the same time interval. Figure 16 and Figure 17 present the body acceleration and wheel–ground contact-force responses, respectively, while Table 6 and Table 7 summarize the corresponding RMS values.

Figure 16 compares the body vertical acceleration of the conventional suspension and the inerter suspension on rough mare and rough upland terrains. In both cases, the acceleration fluctuates strongly due to the uneven lunar surface. For the rough mare terrain, the conventional suspension shows larger acceleration peaks and stronger vibration. The inerter suspension reduces the fluctuation amplitude and suppresses several transient peaks, indicating better vibration isolation. For the rough upland terrain, the excitation is more irregular and severe. The inerter suspension still maintains a relatively smaller acceleration response than the conventional suspension. These results indicate a lower body-vibration response for the inerter suspension under both investigated terrain inputs. 

Figure 17 shows the wheel–ground contact force under the two terrain conditions. A contact force of zero indicates wheel lift-off. On the rough mare terrain, the conventional suspension produces larger force fluctuations and higher impact peaks. The inerter suspension gives a smoother contact force response and reduces peak loads, which helps improve wheel–ground contact stability. On the rough upland terrain, the contact force varies more sharply, and several zero-force moments appear, indicating temporary wheel lift-off. Compared with the conventional suspension, the inerter suspension reduces contact-force fluctuations and peak loads under the investigated terrain inputs.

**Figure 18:**
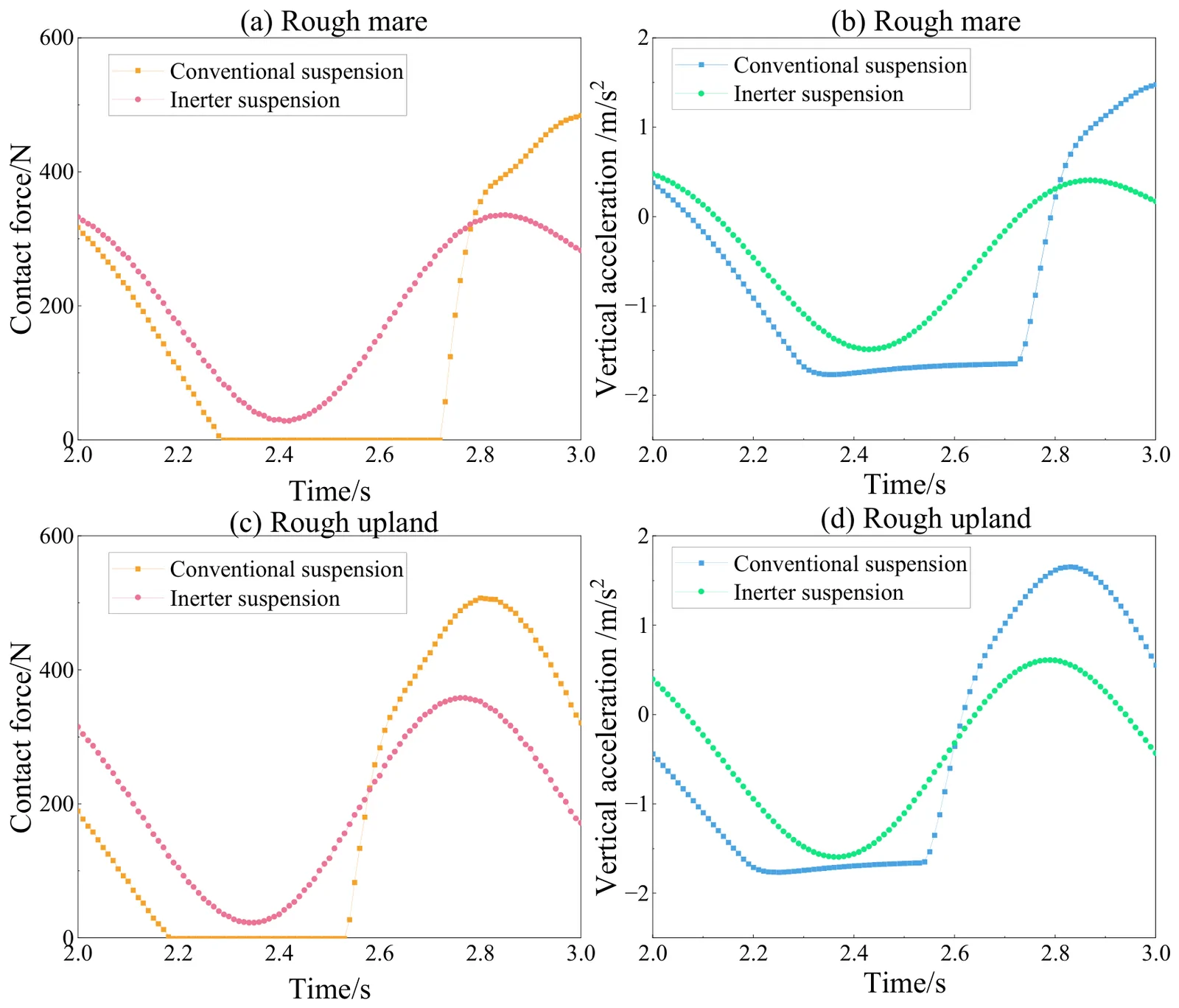
Enlarged comparison from 2 to 3 s: (**a**) wheel–ground contact force and (**b**) body vertical acceleration under rough-mare excitation; (**c**) wheel–ground contact force; and (**d**) body vertical acceleration under rough-upland excitation.

Figure 18 compares the wheel–ground contact force and vehicle-body acceleration from 2 s to 3 s under two lunar surface excitations. The conventional suspension shows wheel lift-off in some intervals, where the contact force drops to zero. In contrast, the inerter suspension maintains a positive contact force, indicating improved wheel–ground contact stability. Meanwhile, the inerter suspension reduces the peak value and fluctuation of the vertical acceleration compared with the conventional suspension. Over this interval, the inerter suspension maintains wheel–ground contact while reducing the peaks and fluctuations of body acceleration.

The comparison of the RMS values of vibration performance in Table 6 indicates that, under rough mare excitation, the inerter suspension system of the manned lunar rover reduced the RMS value of the vehicle body acceleration by 19.61% and the RMS value of the wheel–ground contact force by 13.75% compared to the conventional suspension system. The comparison of the RMS values of vibration performance in Table 7 reveals that, under rough upland excitation, the inerter suspension system of the manned lunar rover reduced the RMS value of the vehicle body acceleration by 15.66% and the RMS value of the wheel–ground contact force by 13.67% compared to the conventional suspension system.

**Figure 19:**
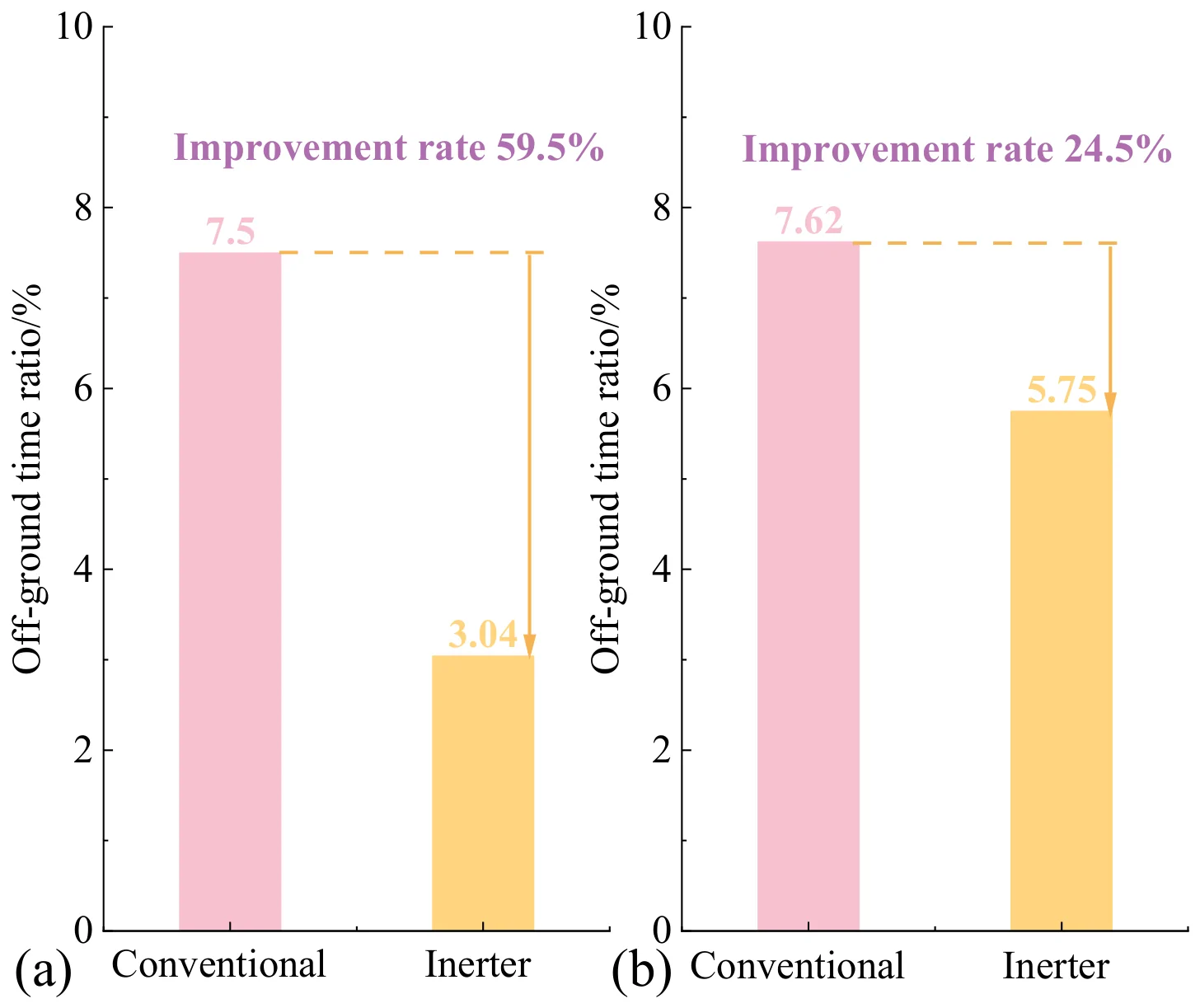
Comparison of lift-off time ratio: (**a**) rough mare; (**b**) rough upland.

**Figure 20:**
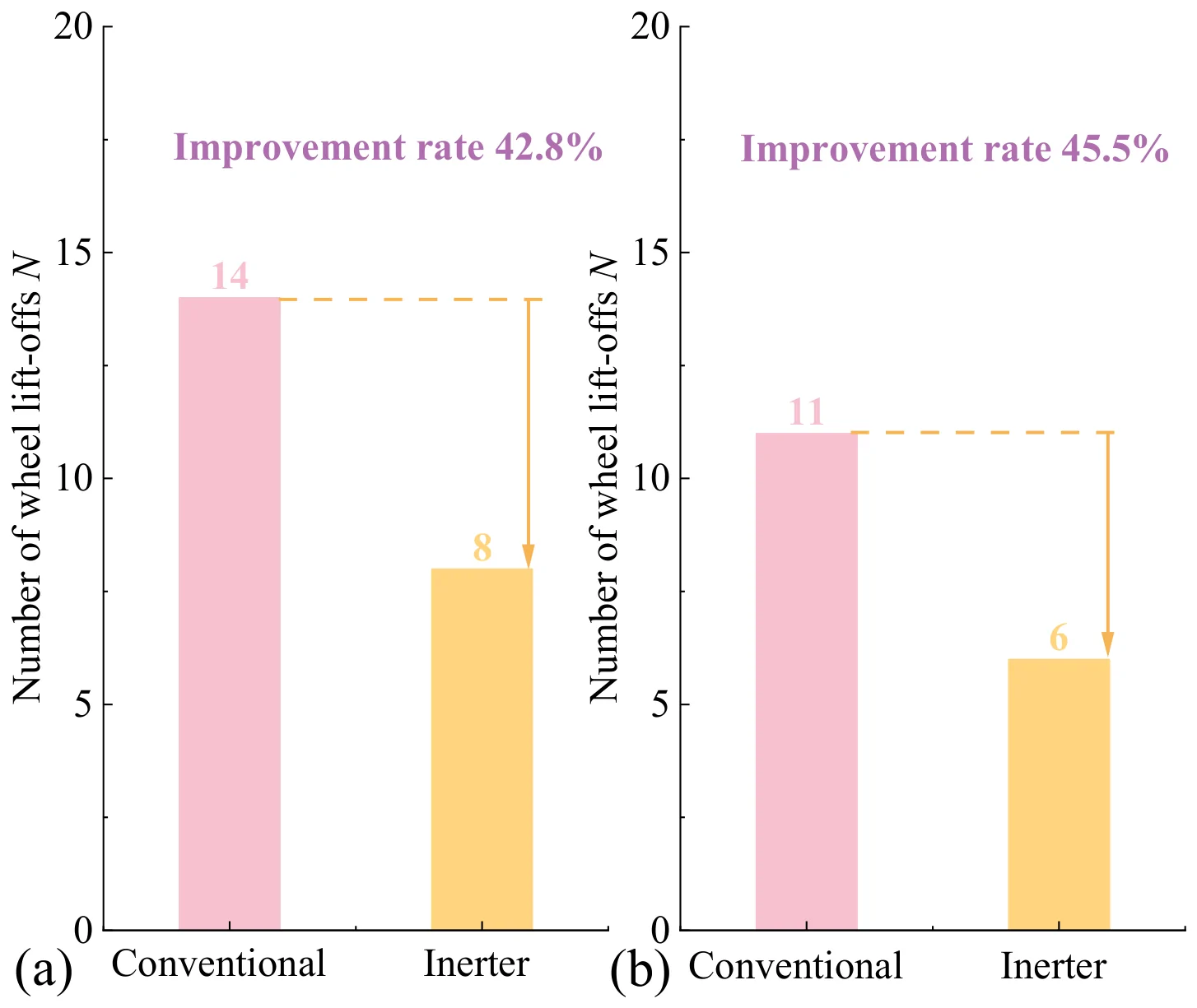
Comparison of number of wheel lift-offs: (**a**) rough mare; (**b**) rough upland.

Wheel–ground contact retention is further evaluated using the off-ground time ratio and the number of wheel lift-offs, as shown in Figure 19 and Figure 20. The off-ground time ratio is estimated as the percentage of uniformly spaced samples for which the normal contact force is zero over the evaluation interval. A wheel lift-off is counted when the normal contact force changes from a positive value to zero, with consecutive zero-force samples belonging to the same separation event.

Under rough-mare excitation, the optimized suspension reduces the off-ground time ratio from 7.50% to 3.04% and the number of wheel lift-offs from 14 to 8, corresponding to reductions of 59.5% and 42.8%, respectively. Under rough-upland excitation, the off-ground time ratio decreases from 7.62% to 5.75%, while the number of wheel lift-offs decreases from 11 to 6, corresponding to reductions of 24.5% and 45.5%, respectively. For both terrain inputs, the optimized suspension exhibits a lower proportion of time in separation and fewer lift-off events.

Taken together, the acceleration, contact-force, and lift-off results indicate that the optimized series inerter suspension attenuates body vibration and improves wheel–ground contact retention under the two investigated terrain excitations.

### 4.4. Robustness Assessment Under Terrain Excitation Variations

An additional rough-mare profile was generated using the method in Section 4.1. An amplitude scaling factor of 1.00 denotes the baseline amplitude of this newly generated profile, while 1.20 denotes a 20% increase in its displacement amplitude. The two optimized parameter sets in Table 5 were held fixed. At each amplitude level, all suspension cases used identical terrain inputs and initial conditions at 3 m/s, with performance evaluated over 0–60 s. 

**Figure 21:**
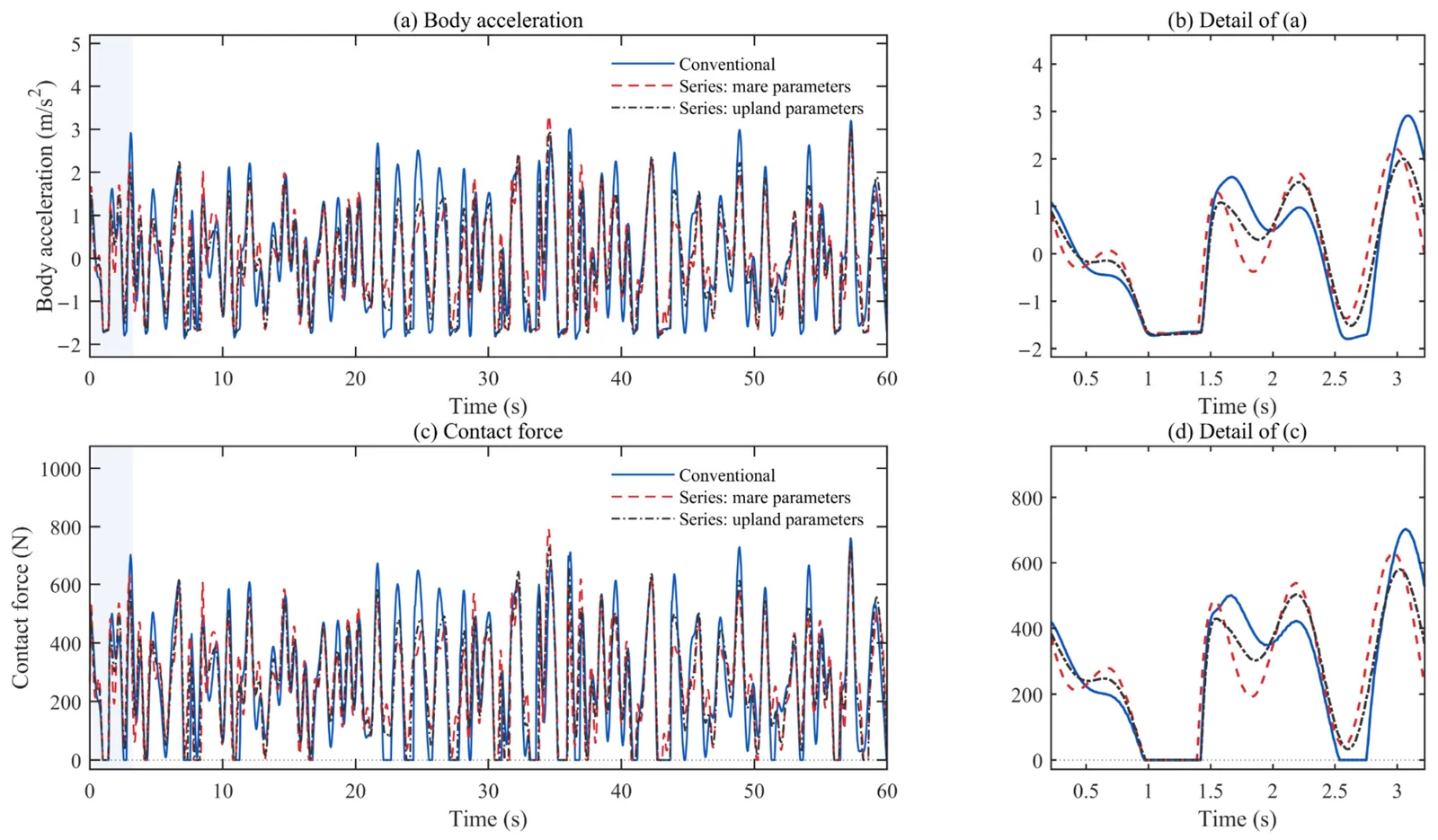
Suspension responses under rough-mare excitation with a 20% amplitude increase: (**a**) body acceleration; (**b**) enlarged acceleration response; (**c**) contact force; (**d**) enlarged contact-force response.

Figure 21 compares the responses at an amplitude scaling factor of 1.20. At approximately 2.54–2.75 s, the conventional suspension loses wheel–ground contact, while both series-suspension cases maintain positive contact forces. Around 3 s, both series-suspension cases also exhibit lower body-acceleration and contact-force peaks.

Table 8 summarizes the results at both amplitude levels. Series-M and Series-U denote the series suspension using the rough-mare and rough-upland optimized parameter sets in Table 5, respectively.

Both optimized parameter sets retain advantages in vibration reduction and contact retention at the two evaluated amplitude levels. The relative reduction in off-ground time ratio becomes smaller as the excitation amplitude increases, indicating that the extent of the improvement depends on excitation severity.

## 5. Conclusions

This study investigated how inerter–damper topology and parameter matching affect vertical vibration and wheel–ground contact retention under lunar gravity. Using a dimensionless piecewise quarter-vehicle model, the effects of wheel–ground separation were examined, and parallel and series configurations were compared through frequency-response, lift-off-boundary, and time-domain analyses.

The frequency-response and lift-off-boundary analyses showed that wheel–ground separation affects the predicted vertical response, highlighting the importance of accounting for contact-state changes in suspension analysis. For the parameter combinations examined, the series configuration provided a larger wheel–ground contact region and better lift-off suppression than the parallel configuration and was therefore selected for parameter optimization.

Under the rough-mare and rough-upland excitations, the optimized series inerter–damper suspension reduced the simulated body acceleration and improved wheel–ground contact retention compared with the conventional suspension. Compared with the conventional suspension, the RMS body acceleration was reduced by 19.61% and 15.66%, and the RMS wheel–ground contact force was reduced by 13.75% and 13.67%, respectively. In addition, the off-ground time ratio was reduced by up to 59.5%, and the number of wheel lift-offs was reduced by up to 45.5%.

These results demonstrate the potential of the series inerter–damper configuration to improve vertical vibration performance and wheel–ground contact retention under the investigated lunar-terrain conditions. The optimized parameter sets and the reported improvement rates correspond to the prescribed terrain inputs, vehicle parameters, and optimization objectives. The reported improvements are simulation-based predictions within the assumptions of the present model. The numerical verification supports computational consistency, while the physical accuracy of the model remains to be assessed against independent experimental measurements. A limitation of the present study is that the quarter-vehicle model describes the vertical response at a single wheel position and does not capture full-rover pitch and roll or load redistribution among the wheels. These coupled effects may alter the onset and duration of wheel lift-off and consequently affect the quantitative improvements obtained at the full-rover level. Future work will assess the transferability of the optimized parameters across terrain conditions and vehicle-parameter variations, together with experimental verification and extension to full-vehicle lunar-rover dynamics.

## Figures and Tables

**Table 1:** Mechanical parameters of lunar surface regolith.

Type	Symbol	Value	Unit
Sinkage exponent	*n*	1.0	/
Cohesive deformation modulus	$K_{c}$	1.4	${\mathrm{kPa}/m}^{n-1}$
Frictional deformation modulus	$K_{\varphi }$	820	${\mathrm{kPa}/m}^{n}$

**Table 2:** Dimensionless parameters.

Parameters	ε	α	ξ	β	$Y_{0}$	γ
Earth value	0.1	5.7554	0.3856	0	0.6185	[0, 20]
Lunar surface value for parallel configuration	0.1	5.7554	0.3856	0.1/0.2/0.3	3.7117	[0, 20]
Lunar surface value for series configuration	0.1	5.7554	0.3856	0.8/1.0/1.2	3.7117	[0, 20]

Here, ε, α, ξ, β, $Y_{0}$ and γ denote the mass ratio, stiffness ratio, damping ratio, inertance ratio, dimensionless excitation amplitude, and frequency ratio, respectively. The dimensional road-displacement amplitude $y_{0}=0.06\;m$ is prescribed for both Earth and lunar gravity. The dimensionless excitation amplitudes are calculated separately using the gravity-dependent characteristic length.

**Table 3:** Response errors and off-ground time fractions for the six verification cases.

Suspension Configuration	Y	β	$E_{a}(\%)$	$E_{N}(\%)$	$\eta_{off}^{DE}(\%)$	$\eta_{off}^{MN}(\%)$
Conventional	3.7117	0	7.474 × 10^−5^	7.063 × 10^−5^	24.4925	24.4925
Parallel	3.7117	0.2	2.824 × 10^−6^	8.871 × 10^−6^	0	0
Series	3.7117	0.8	2.045 × 10^−5^	2.807 × 10^−5^	0	0
Conventional	7.4234	0	3.136 × 10^−3^	4.189 × 10^−3^	50.9525	50.9525
Parallel	7.4234	0.2	5.118 × 10^−4^	6.382 × 10^−4^	48.4325	48.4325
Series	7.4234	0.8	2.255 × 10^−4^	2.829 × 10^−4^	31.2575	31.2625

Note: $E_{a}$ and $E_{N}$ denote the range-normalized root-mean-square errors in dimensionless sprung-mass acceleration and normalized normal force, respectively. DE and MN denote the direct-equation and mechanical-network formulations, respectively. The off-ground time fraction, $\eta_{off}$, was evaluated over τ=20−60 by trapezoidal integration of the indicator $F_{N}/W_{0}\leq 10^{-10}$ on a common time grid with a spacing of △τ=0.002.

**Table 4:** Optimized parameters and optimization range.

Parameters	Description	Range
b/kg	Inertance coefficient	[0, 800]
c/N·s/m	Damping coefficient	[0, 6000]

**Table 5:** Optimal parameter combination.

Excitation	b (kg)	c (N·s/m)
Rough mare	228.63	933.28
Rough upland	229.14	615.46

**Table 6:** The RMS values of the vibration performance under rough mare excitation.

	Body Acceleration RMS (m/s^2^)	Wheel–Ground Contact Force RMS (N)
Conventional	1.0192	307.9344
Inerter suspension	0.8193	265.5881
Improvement rate	19.61%	13.75%

**Table 7:** The RMS values of the vibration performance under rough upland excitation.

	Body Acceleration RMS (m/s^2^)	Wheel–Ground Contact Force RMS (N)
Conventional	0.9083	293.0379
Inerter suspension	0.7661	252.9925
Improvement rate	15.66%	13.67%

**Table 8:** Suspension performance at different excitation amplitudes.

Amplitude Factor	Suspension	RMS Body Acceleration $\left(m/s^{2}\right)$	RMS Contact Force (N)	Off-Ground Time Ratio (%)	Number of Wheel Lift-Offs
1.00	Conventional	1.0811	313.1231	8.13	12
1.00	Series-rough mare parameters	0.8763	300.4386	3.74	7
1.00	Series-rough upland parameters	0.9290	302.4860	4.15	8
1.20	Conventional	1.2889	331.4176	14.70	22
1.20	Series-rough mare parameters	1.0525	314.5679	9.09	15
1.20	Series-rough upland parameters	1.1138	317.4319	10.35	17

## Data Availability

The data presented in this study are available on request from the corresponding author. The data are not publicly available due to privacy or ethical restrictions but may be shared for research purposes upon reasonable request.

## Appendix A

The detailed derivation of the undetermined coefficients $A_{1,i}$, $B_{1,i}$, $A_{2,i}$ and $B_{2,i}$ in Section 2.3.

(1) Parallel inerter–damper configuration

The road excitation is taken as a cosine function:

(A1) $$Z_{r}=Y_{0}\cos\left(\gamma \tau \right)$$

where $\gamma =\frac{\omega }{\omega_{A}}$ is the non-dimensional excitation frequency, and ω is the circular frequency of the road excitation.

Then $Z_{s}$, $Z_{u}$ can be expressed as:

(A2) $$Z_{s}=A_{1,p}\sin(\gamma \tau )+B_{1,p}\cos(\gamma \tau )=\left|Z_{s}\right|\sin(\gamma \tau +\phi_{s})$$

(A3) $$Z_{u}=A_{2,p}\sin(\gamma \tau )+B_{2,p}\cos(\gamma \tau )=\left|Z_{u}\right|\sin(\gamma \tau +\phi_{u})$$

where $\phi_{s}$ represents the phase of the spring-mass displacement, and $\phi_{u}$ represents the phase of the unsprung-mass displacement.

The undetermined coefficient $A_{1,p}$, $B_{1,p}$, $A_{2,p}$, $B_{2,p}$ can be solved using the following equation:

(A4) $$\left[A\right]\left[\begin{array}{c} A_{1,p}\\ B_{1,p}\\ A_{2,p}\\ B_{2,p} \end{array}\right]=\left[\begin{array}{c} 0\\ 0\\ 0\\ \alpha Y_{0} \end{array}\right]$$

where:

(A5) $$[A]=\left[\begin{array}{cccc} P & -2\xi \gamma & Q & 2\xi \gamma \\ 2\xi \gamma & P & -2\xi \gamma & Q\\ Q & 2\xi \gamma & R & -2\xi \gamma \\ -2\xi \gamma & Q & 2\xi \gamma & R \end{array}\right]$$

The solutions are:

(A6) $$A_{1,p}=\frac{-\alpha Y_{0}\left(QD_{2}+2\xi \gamma D_{1}\right)}{{D_{1}}^{2}+{D_{2}}^{2}}$$

(A7) $$B_{1,p}=\frac{\alpha Y_{0}\left(-QD_{1}+2\xi \gamma D_{2}\right)}{{D_{1}}^{2}+{D_{2}}^{2}}$$

(A8) $$A_{2,p}=\frac{\alpha Y_{0}\left(PD_{2}-2\xi \gamma D_{1}\right)}{{D_{1}}^{2}+{D_{2}}^{2}}$$

(A9) $$B_{2,p}=\frac{\alpha Y_{0}\left(PD_{1}+2\xi \gamma D_{2}\right)}{{D_{1}}^{2}+{D_{2}}^{2}}$$

where:

(A10) $$P=1-(1+\beta )\gamma^{2}$$

(A11) $$Q=-1+\beta \gamma^{2}$$

(A12) $$R=(1+\alpha )-(\varepsilon +\beta )\gamma^{2}$$

(A13) $$D_{1}=PR-Q^{2}$$

(A14) $$D_{2}=2\xi \gamma (P+R+2Q)$$

Therefore, the amplitude of the system’s steady-state response at the point of wheel–ground contact is:

(A15) $${\left|Z_{s}\right|}^{2}={A_{1,p}}^{2}+{B_{1,p}}^{2}=\frac{\alpha^{2}{Y_{0}}^{2}\left(Q^{2}+4\xi^{2}\gamma^{2}\right)}{{D_{1}}^{2}+{D_{2}}^{2}};\left|Z_{s}\right|=\sqrt{{A_{1,p}}^{2}+{B_{1,p}}^{2}}$$

(A16) $${\left|Z_{u}\right|}^{2}={A_{2,p}}^{2}+{B_{2,p}}^{2}=\frac{\alpha^{2}{Y_{0}}^{2}\left(P^{2}+4\xi^{2}\gamma^{2}\right)}{{D_{1}}^{2}+{D_{2}}^{2}};\left|Z_{u}\right|=\sqrt{{A_{2,p}}^{2}+{B_{2,p}}^{2}}$$

(A17) $$\tan\phi_{s}=\frac{B_{1,p}}{A_{1,p}}\;\;\;\;\tan\phi_{u}=\frac{B_{2,p}}{A_{2,p}}$$

(A18) $$Z_{s}=A_{1,p}\sin(\gamma \tau )+B_{1,p}\cos(\gamma \tau )=\left|Z_{s}\right|\sin(\gamma \tau +\phi_{s})$$

(A19) $$Z_{u}=A_{2,p}\sin(\gamma \tau )+B_{2,p}\cos(\gamma \tau )=\left|Z_{u}\right|\sin(\gamma \tau +\phi_{u})$$

The wheel–ground clearance $Z_{t}$ is as follows:

(A20) $$Z_{t}=Z_{u}-Z_{r}=A_{2,p}\sin(\gamma \tau )+(B_{2,p}-Y_{0})\cos(\gamma \tau )$$

(A21) $$\begin{array}{l} {\left|Z_{t}\right|}^{2}={A_{2,p}}^{2}+{\left(B_{2,p}-Y_{0}\right)}^{2}\\ \;\;\;\;\;\;={Y_{0}}^{2}\left(\frac{\alpha^{2}(P^{2}+4\xi^{2}\gamma^{2})}{{D_{1}}^{2}+{D_{2}}^{2}}-\frac{2\alpha (PD_{1}+2\xi \gamma D_{2})}{{D_{1}}^{2}+{D_{2}}^{2}}+1\right) \end{array}$$

(A22) $$\left|Z_{t}\right|=\sqrt{{A_{2,p}}^{2}+{\left(B_{2,p}-Y_{0}\right)}^{2}}$$

(2) Series inerter–damper configuration

(A23)
$$Z_{rs}=Y_{0}\cos(\gamma \tau )$$

(A24)
$$Z_{ss}=A_{1,s}\sin(\gamma \tau )+B_{1,s}\cos(\gamma \tau )=\left|Z_{ss}\right|\sin(\gamma \tau +\phi_{ss})$$

(A25)
$$Z_{us}=A_{2,s}\sin(\gamma \tau )+B_{2,s}\cos(\gamma \tau )=\left|Z_{us}\right|\sin(\gamma \tau +\phi_{us})$$

(A26)
$$Z_{bs}=A_{3,s}\sin(\gamma \tau )+B_{3,s}\cos(\gamma \tau )=\left|Z_{bs}\right|\sin(\gamma \tau +\phi_{bs})$$

(A27)
$$F_{s}=A_{4,s}\sin(\gamma \tau )+B_{4,s}\cos(\gamma \tau )=\left|F_{s}\right|\sin(\gamma \tau +\phi_{F})$$

By solving the equations simultaneously in Formula (29) and eliminating $Z_{bs}$ and $F_{s}$, $A_{3,s}$, $B_{3,s}$, $A_{4,s}$, $B_{4,s}$ can be obtained.

(A28) $$A_{3.s}=A_{1,s}+\frac{A_{4,s}}{\beta \gamma^{2}}\;\;\;\;\;\;\;\;B_{3,s}=B_{1,s}+\frac{B_{4,s}}{\beta \gamma^{2}}$$

(A29) $$\begin{array}{l} A_{4,s}=-\frac{\frac{1}{2\xi \gamma }\left(B_{1,s}-B_{2,s}\right)+\frac{1}{\beta \gamma^{2}}\left(A_{1,s}-A_{2,s}\right)}{S}\;\\ B_{4,s}=\frac{\frac{1}{2\xi \gamma }\left(A_{1,s}-A_{2,s}\right)-\frac{1}{\beta \gamma^{2}}\left(B_{1,s}-B_{2,s}\right)}{S} \end{array}$$

(A30) $$S={\left(\frac{1}{2\xi \gamma }\right)}^{2}+{\left(\frac{1}{\beta \gamma^{2}}\right)}^{2}$$

The remaining undetermined real coefficient $A_{1,s}$, $B_{1,s}$, $A_{2,s}$, $B_{2,s}$ can be solved using the following relationship:

(A31) $$\left[B\right]\left[\begin{array}{c} A_{1,s}\\ B_{1,s}\\ A_{2,s}\\ B_{2,s} \end{array}\right]=\left[\begin{array}{c} 0\\ 0\\ 0\\ \alpha SY_{0} \end{array}\right]$$

where:

(A32) $$B=\left[\begin{array}{cccc} P_{1} & -\frac{1}{2\xi \gamma } & Q_{1} & \frac{1}{2\xi \gamma }\\ \frac{1}{2\xi \gamma } & P_{1} & -\frac{1}{2\xi \gamma } & Q_{1}\\ Q_{1} & \frac{1}{2\xi \gamma } & R_{1} & -\frac{1}{2\xi \gamma }\\ -\frac{1}{2\xi \gamma } & Q_{1} & \frac{1}{2\xi \gamma } & R_{1} \end{array}\right]$$

(A33) $$P_{1}=S\left(1-\gamma^{2}\right)-\frac{1}{\beta \gamma^{2}}$$

(A34) $$Q_{1}=-S+\frac{1}{\beta \gamma^{2}}$$

(A35) $$R_{1}=S\left(-\varepsilon \gamma^{2}+\alpha +1\right)-\frac{1}{\beta \gamma^{2}}$$

The solutions are:

(A36) $$A_{1,s}=\frac{-\alpha SY_{0}\left(Q_{1}S_{2}+\frac{1}{2\xi \gamma }S_{1}\right)}{{S_{1}}^{2}+{S_{2}}^{2}}$$

(A37) $$B_{1,s}=\frac{\alpha SY_{0}\left(-Q_{1}S_{1}+\frac{1}{2\xi \gamma }S_{2}\right)}{{S_{1}}^{2}+{S_{2}}^{2}}$$

(A38) $$A_{2,s}=\frac{\alpha SY_{0}\left(P_{1}S_{2}-\frac{1}{2\xi \gamma }S_{1}\right)}{{S_{1}}^{2}+{S_{2}}^{2}}$$

(A39) $$B_{2,s}=\frac{\alpha SY_{0}\left(P_{1}S_{1}+\frac{1}{2\xi \gamma }S_{2}\right)}{{S_{1}}^{2}+{S_{2}}^{2}}$$

(A40) $$S_{1}=P_{1}R_{1}-{Q_{1}}^{2}$$

(A41) $$S_{2}=\frac{1}{2\xi \gamma }\left(P_{1}+R_{1}+2Q_{1}\right)$$

Therefore, $Z_{ss}$, $Z_{us}$ can be expressed as follows:

(A42) $${\left|Z_{ss}\right|}^{2}={A_{1,s}}^{2}+{B_{1,s}}^{2};\;\;\;\;\left|Z_{ss}\right|=\sqrt{{A_{1,s}}^{2}+{B_{1,s}}^{2}}$$

(A43) $$\left|Z_{ss}\right|=\sqrt{{A_{1,s}}^{2}+{B_{1,s}}^{2}}\;\;\;\;\;\;\left|Z_{us}\right|=\sqrt{{A_{2,s}}^{2}+{B_{2,s}}^{2}}$$

(A44) $$\tan\phi_{ss}=\frac{B_{1,s}}{A_{1,s}}\;\;\;\;\tan\phi_{us}=\frac{B_{2,s}}{{A_{2,}}_{s}}$$

(A45) $$Z_{ss}=A_{1,s}\sin(\gamma \tau )+B_{1,s}\cos(\gamma \tau )=\left|Z_{ss}\right|\sin(\gamma \tau +\phi_{ss})$$

(A46) $$Z_{us}=A_{2,s}\sin(\gamma \tau )+B_{2,s}\cos(\gamma \tau )=\left|Z_{us}\right|\sin(\gamma \tau +\phi_{us})$$

The wheel–ground clearance $Z_{ts}$ is as follows:

(A47) $$Z_{ts}=Z_{us}-Z_{rs}=A_{2,s}\sin(\gamma \tau )+(B_{2,s}-Y_{0})\cos(\gamma \tau )$$

(A48) $$\begin{array}{l} {\left|Z_{ts}\right|}^{2}={A_{2,s}}^{2}+{\left(B_{2,s}-Y_{0}\right)}^{2}\\ \;\;\;\;\;={Y_{0}}^{2}\left(\frac{\alpha^{2}S^{2}\left({P_{1}}^{2}+\frac{1}{4\xi^{2}\gamma^{2}}\right)}{{S_{1}}^{2}+{S_{2}}^{2}}-\frac{2\alpha S\left(P_{1}S_{1}+\frac{1}{2\xi \gamma }S_{2}\right)}{{S_{1}}^{2}+{S_{2}}^{2}}+1\right) \end{array}$$

(A49) $$\left|Z_{ts}\right|=\sqrt{{A_{2,s}}^{2}+{\left(B_{2,s}-Y_{0}\right)}^{2}}$$
